# Isolation of Human Milk Difucosyl Nona- and Decasaccharides by Ultrahigh-Temperature Preparative PGC-HPLC and Identification of Novel Difucosylated Heptaose and Octaose Backbones by Negative-Ion ESI-MS^n^

**DOI:** 10.1021/acs.analchem.3c05008

**Published:** 2024-04-15

**Authors:** Cuiyan Cao, Yiming Cheng, Yi Zheng, Beibei Huang, Zhimou Guo, Long Yu, Barbara Mulloy, Virginia Tajadura-Ortega, Wengang Chai, Jingyu Yan, Xinmiao Liang

**Affiliations:** † Dalian Institute of Chemical Physics, Chinese Academy of Sciences, Key Laboratory of Separation Science for Analytical Chemistry, Dalian 116023, China; ‡ Jiangxi Provincial Key Laboratory for Pharmacodynamic Material Basis of Traditional Chinese Medicine, Ganjiang Chinese Medicine Innovation Center, Nanchang 330000, China; § University of Chinese Academy of Sciences, Beijing 100049, China; ∥ Glycosciences Laboratory, Faculty of Medicine, Imperial College London, Hammersmith Campus, London W12 0NN, United Kingdom

## Abstract

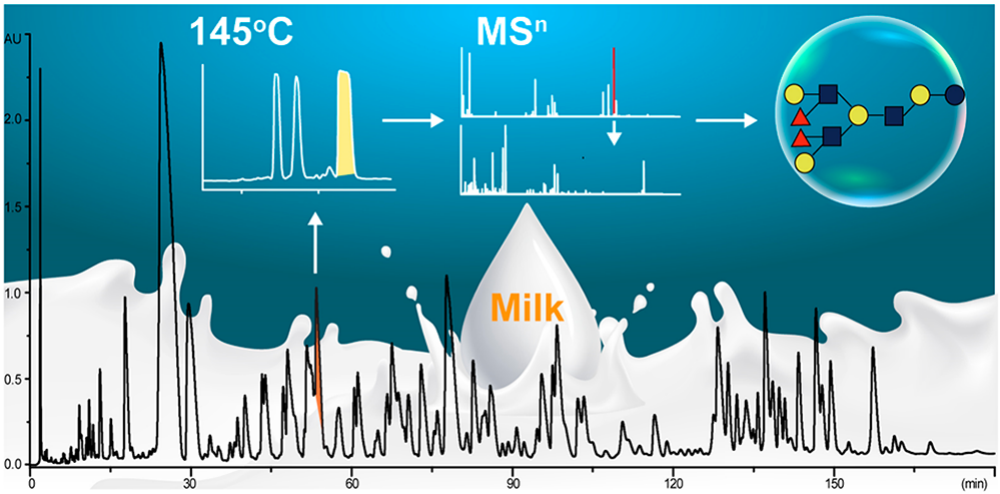

Despite their many important physiological functions, past work on the diverse sequences of human milk oligosaccharides (HMOs) has been focused mainly on the highly abundant HMOs with a relatively low degree of polymerization (DP) due to the lack of efficient methods for separation/purification and high-sensitivity sequencing of large-sized HMOs with DP ≥ 10. Here we established an ultrahigh-temperature preparative HPLC based on a porous graphitized carbon column at up to 145 °C to overcome the anomeric α/β splitting problem and developed further the negative-ion ESI-CID-MS/MS into multistage MS^n^ using a combined product-ion scanning of singly charged molecular ion and doubly charged fragment ion of the branching Gal and adjacent GlcNAc residues. The separation and sequencing method allows efficient separation of a neutral fraction with DP ≥ 10 into 70 components, among which 17 isomeric difucosylated nona- and decasaccharides were further purified and sequenced. As a result, novel branched difucosyl heptaose and octaose backbones were unambiguously identified in addition to the conventional linear and branched octaose backbones. The novel structures of difucosylated DF-*novo*-heptaose, DF-*novo*-LNO I, and DF-*novo*-LNnO I were corroborated by NMR. The various fucose-containing Lewis epitopes identified on different backbones were confirmed by oligosaccharide microarray analysis.

Human milk oligosaccharides (HMOs) make up the third most abundant component and consist of a lactose core decorated with N-acetylglucosamine (GlcNAc), d-galactose (Gal), l-fucose (Fuc), and sialic acid (NeuAc). Fucosylated HMOs are most abundant and form the blood group ABH(O) and Lewis (Le) epitopes.^1,2^ HMOs have been identified as decoy receptors preventing infections by many pathogens and regulate cell surface receptors triggering immune responses.^3−5^ HMOs also play important roles in regulation of gut microbiota,^6,7^ promote the survival of beneficial Bifidobacterium species, and suppress potentially harmful or pathogenic bacteria.^8,9^

Despite their many important physiological functions, past work has been focused mainly on the high-abundance HMOs with a relatively low degree of polymerization (DP) due to the lack of efficient methods for separation/purification and high-sensitivity sequencing. Separation and sequence assignment of the large sized HMOs (e.g., DP ≥ 10) have been difficult^10,11^ because of their hugely diverse and isomeric structural features, particularly the multiply fucosylated structures carrying different recognition motifs on backbones with different branching patterns,^12,13^ in addition to their low abundance (<1% of total HMOs). The slow progress in method development to tackle the problem of high sequence complexity of HMOs has hindered the in-depth understanding of the structure–function relationships of different types of HMOs.

In the recent past, HPLC with a porous graphitic carbon (PGC) column has been widely used for separation/purification of different types of carbohydrate molecules because of its high resolution. However, α/β anomeric forms of reducing glycans are a unique feature of naturally derived sugars and can cause considerable problems for separation/purification. The α/β splitting increases the number of eluted peaks.^14^ In many cases, one component’s α can be merged with another component’s β, and this has been an obstacle for HPLC separation. For analytical LC-MS using single-ion monitoring, this may not be a major problem, but for preparative fractionation to isolate individual oligosaccharides, the splitting problem is a big headache in chromatography. Although chemical reduction can eliminate the α/β splitting,^15^ it is not ideal for subsequent mass spectrometry (MS) sequencing as some of the well-established methods for neutral sugars require reducing termini, as some characteristic fragmentations observed for typing of blood group and Lewis antigens,^16^ branching pattern,^17^ and partial linkage^18^ analysis can only be obtained for reducing sugars with the hemiacetal functionality but not for reduced alditols.^19^ Moreover, the alditols cannot be used for reducing terminal tagging, a strategy conventionally employed for high-sensitivity HPLC and activity detection by glycan microarrays after their conversion into glycan probes.^20^ As PGC is stable under some extreme conditions (e.g., high and low pH, and high temperature) without any loss in performance,^21,22^ ammonium hydroxide has been added to alleviate the anomeric problem.^23^ Recently, ultrahigh column temperature has been attempted to circumvent the α/β splitting by acceleration of the interconversion between α and β anomers, and satisfactory results were obtained for N-glycans using analytical scale UPLC-MS.^24^ However, preparative scale HPLC at a high temperature has not been attempted. Preparative HPLC is important for the isolation and preparation of reducing glycans from natural sources for functional glycomics.

For glycan structural analysis, MS has become the major player.^16,17,25−27^ Negative-ion electrospray tandem MS with collision-induced dissociation (ESI-CID-MS/MS) has been developed for the high-sensitivity sequencing of HMOs. However, for multifucosylated and highly branched HMOs with high DPs, MS^2^ is not sufficient while quasi-MS^3^ is not always achievable^25^ for assignment of branched sequences. The lack of direct sequence information on the 3-branch remains a problem.^17^

In the present work, we aimed to develop methods to tackle both separation and sequencing problems. A preparative-scale PGC-HPLC using ultrahigh temperature (UHT) is developed for separation and purification of isomeric multifucosylated HMOs in the deca- and dodecasaccharide range, and off-line negative-ion multistage product-ion scanning ESI-CID-MS^n^ is established for branching pattern analysis and detailed assignment of the difucosylated neutral HMOs on different backbones. As a result, novel heptaose and octaose backbones with different fucosylation patterns were identified. The novel structural features were corroborated by NMR and peripheral epitopes, which were corroborated by microarray analysis.

## Experimental Section

### Materials

All solvents were of HPLC grade, and other reagents used were of analytical grade or higher. Human breast milk samples (∼20 L) were collected from healthy mothers at different lactation periods and stored at −20 °C before use. Oligosaccharide standards lactose-N-fucopentaose (LNFP) I, II, III, 3′-sialyllactose (3′-SL) and 6′-sialyllactose (6′-SL) were purchased from Dextra Laboratories (Reading, England). All the carbohydrate-binding proteins were purchased from commercial sources as listed in Supplementary Methods.

### Fucosylated Decasaccharide Fraction from Human Milk

Pretreatment of HMOs was as described previously.^28^ The total HMOs was subjected to preparative HILIC on a Click TE-GSH column (100 × 250 mm, 5 μm, Acchrom) to remove the large amount of lactose and separate neutral from sialylated oligosaccharides.^29^ The pooled fraction F5 containing neutral HMOs with DP ≥ 10 was then further fractionated on a preparative amide column (100 × 250 mm, 5 μm, Acchrom) to obtain the “DP10” fraction (Supplementary Methods) which was then subfractionated as described below.

### Preparative-Scale Ultrahigh-Temperature PGC-HPLC of HMOs

A preparative-scale UHT HPLC system was built based on a Waters Alliance HPLC system with a UV photodiode array detector 2998. A purpose built high-temperature oven, postcolumn cooling device, and high-temperature and cooling controllers were added into the system as modular components (Figure S1a). The high-temperature oven contains an aluminum shell heater and insulation plates wrapped by aluminum alloy heat sink with a maximum operating temperature of at least 180 °C (Figure S1b). The postcolumn cooling device consists of a Peltier thermoelectrical cooler module, an aluminum alloy heat sink, and a fan (Figure S1c). For fast reaching and better maintaining of column temperature, a column with shorter diameter is always preferable, although the oven can hold columns with larger diameters.

Initial separation of “DP10” fraction was carried out at 105 °C on a PGC column (4.6 × 150 mm, 5 μm) with the following solvent gradient: 0–120 min, 10/90 to 25/75 (CH_3_CN/H_2_O); 120–180 min, 25/75 to 50/50 (CH_3_CN/H_2_O), at a flow rate of 1.0 mL/min. The injection volume was 20 μL at a flow rate of 250 μg/μL (5 mg). Seventy fractions were manually collected based on 195 nm detection and concentrated and dried by lyophilization.

Twenty-four fractions containing difucosylated nona- and decasaccharides were further purified on the same column at a flow rate of 0.5 mL/min with an optimized gradient. For some selected fractions, a PGC HT column (3.0 × 100 mm, 3 μm) was used at a column temperature of 145 °C and a flow rate of 0.4 mL/min. The elution was by CH_3_CN/H_2_O gradient with or without 0.1% formic acid and monitored at 195 nm.

The purity of the isolated HMOs was assessed on a PGC HT column at 145 °C and a CH_3_CN/H_2_O gradient of 15/85 to 50/50 (v/v) in 70 min at a flow rate of 0.4 mL/min. The stabilities of neutral and sialylated HMOs under the UHT conditions were examined by HPLC and MS analysis using LNFP-I, 3′-SL, and 6′-SL as examples. The test samples were subjected to the 145 °C PGC-HPLC, and the collected samples were analyzed by PGC-HPLC at 40 °C and ESI-MS.

### Negative-Ion ESI-MS^n^

Negative-ion ESI-MS was performed on an Orbitrap ID-X Tribrid mass spectrometer (Thermo Fisher Scientific) equipped with a Vanquish UHPLC system (Thermo Fisher Scientific). The purified samples were dissolved in CH_3_CN/H_2_O (1:1) at a concentration of 1 μg/μL, of which 2 μL was injected for MS^n^. CH_3_CN/H_2_O (1:1) was used as the mobile phase, with a flow rate of 0.2 mL/min. The spray voltage was at 3.0 kV with a source temperature of 400 °C, ion transfer tube temperature 300 °C, RF S-lens 50 V, and sheath velocity 40 psi. Higher-energy collisional dissociation was used for the MS^n^. For optimal fragmentation, normalized collision energy was adjusted to 15–30% . Precursor selection for product-ion scanning was made manually using the Xcalibur software Version 4.2 data system.

### NMR Spectroscopy

Oligosaccharide samples were submitted to two cycles of dissolution in D_2_O followed by lyophilization to reduce H_2_O content, then taken up in 300 μL of D_2_O and transferred to a Shigemi tube for NMR spectroscopy. NMR spectra were recorded at 950 MHz (1D ^1^H and 2D HSQC, HSQC-TOCSY, HMBC, and H2BC) and 700 MHz (for the ROESY spectra) at 25 °C, using Bruker Avance spectrometers. Pulse sequences were supplied by the spectrometer manufacturer. Chemical shifts are relative to acetone at 2.218 ppm for proton and 33.0 ppm for carbon spectra.

### Oligosaccharide Microarrays

The HMO probes were prepared by reductive-amination with a fluorescent amino-terminating bifunctional linker BABI (J. Yan, Y. Zheng and colleagues, unpublished) and printed in quadruplicate on NHS glass slides (Schott Nexterion H, Germany) at probe concentrations of 25 and 50 μM. After blocking and washing, the arrays were overlaid with biotinylated plant lectins and anticarbohydrate antibodies before incubation at ambient temperature for 90 min. The binding signals were detected using AlexaFluor-647-labeled streptavidin or the biotinylated anti-Mouse IgG with subsequent AlexaFluor-647-labeled streptavidin. Anti-B antibody was detected with antimouse IgM AlexaFluor-680. The fluorescence binding signals were measured and quantified using GenePix Pro 7 software (Molecular Devices). The detailed experimental conditions are in Supplementary Methods.

## Results and Discussion

### Optimization of Conditions for PGC-HPLC Separation of HMO “DP10” Fraction

Using a standard HMO mixture containing LNFP I/II/III, we assessed several conditions to suppress the α/β splitting, including alkaline condition of the mobile phase^23^ and high column temperatures (80 and 110 °C).^24^ As shown in Figure S2a, α- and β-anomers of LNFP I/II/III were well separated at 30 °C although the α-anomer of LNFP III and β-anomer of LNFP II overlapped. Change to alkaline condition (e.g., 0.4% NH_4_OH) improved separation of the three isomers, but the peak shape became very broad, which is not ideal for separation of complex mixtures (Figure S2b). Increasing column temperature to 80 °C (Figure S2c) or 105 °C (Figure S2d) can largely eliminate the α/β splitting while maintaining the good resolution, indicating that higher temperature is necessary for separation of more complex and/or higher oligomeric HMOs.

A UHT system for preparative-scale HPLC was then designed, purpose-built (Figure S1), and tested at 145 °C for resolution of isomeric HMOs in the “DP10” fraction. As shown in Figure S3a, a single peak of decasaccharide collected at 30 °C showed a shoulder at 105 °C, while at 145 °C a second peak with baseline resolution was obtained (Figure S3b, sequence assignment in sections below).

Using LNFP I, 3′-SL, and 6′-SL as examples, we examined the stabilities of HMOs under the UHT conditions. We found that the neutral HMOs are stable (Figure S4). Although sialic acid residues are labile, our experiments indicated that under UHT conditions they are reasonably stable at 145 °C in the HPLC elution time scale (Figure S4a). Even under acidic conditions (e.g., with 0.1% formic acid), a majority of the 6-linked 6′-SL can survive the high temperature but 3-linked 3′-SL cannot (Figure S4b). The results obtained from the sialylated oligosaccharides are different from a previous publication.^24^

### Preparative PGC-HPLC at Ultrahigh Temperature for Fractionation and Purification of Human Milk Nona- and Decasaccharides

After removal of protein and lipid, the total HMOs was subjected to the first dimension HPLC using a Click-TE GSH column with mixed hydrophilic and charge exchange mechanism^29^ to separate the sialylated (F1, Figure S5a) HMOs and the large amount of lactose (F2) and to obtain 3 neutral fractions F3–F5 with increasing oligomeric size. F5 containing the largest HMOs was then further fractionated into 8 pools primarily based on DPs (DP5 to DP12–14, Figure S5b) using an amide HILIC as the second dimension HPLC.

**Figure 1:**
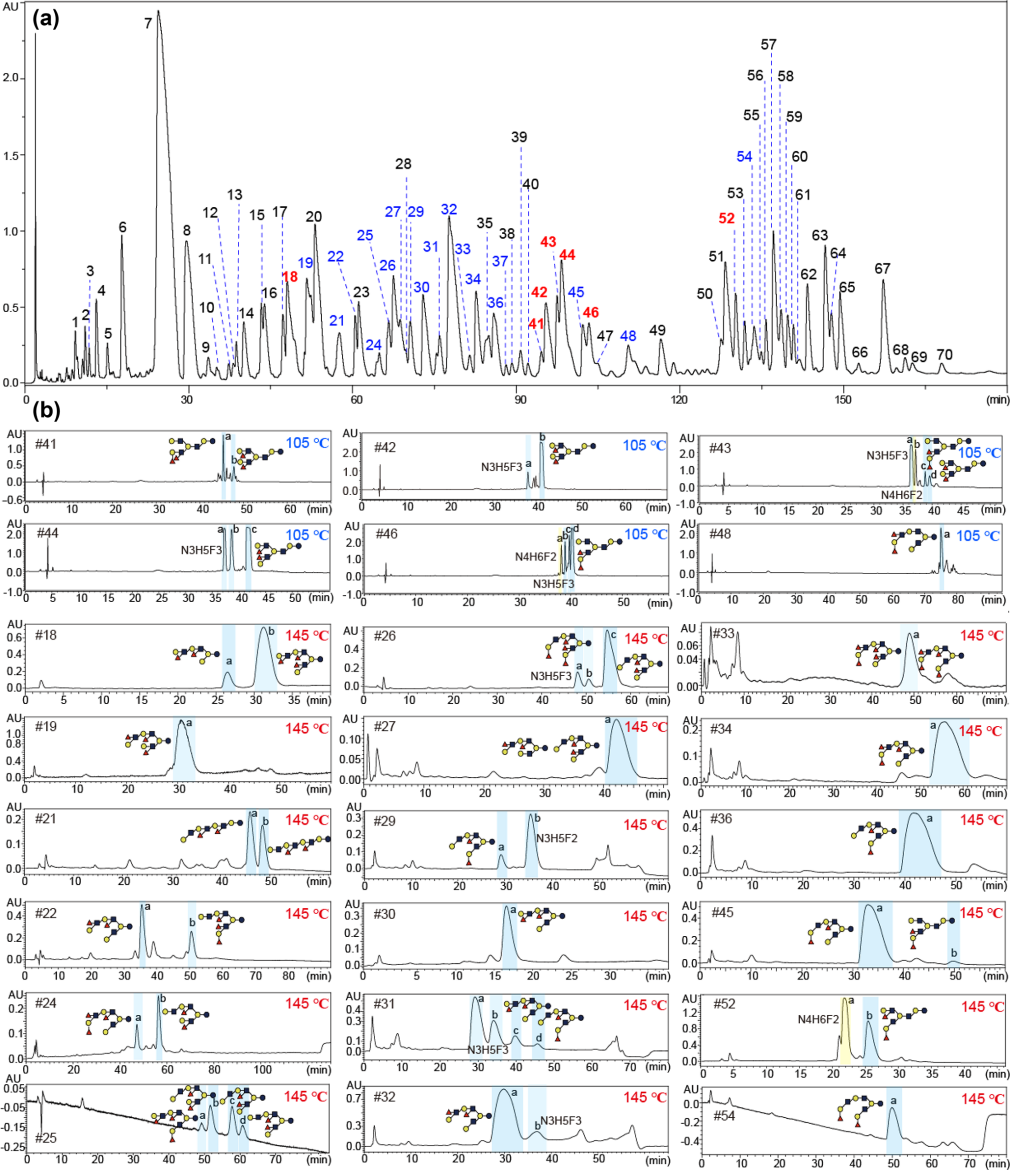
Ultrahigh temperature PGC-HPLC fractionation of difucosylated HMOs with different octaose backbones. (a) PGC-HPLC of the “DP10” fraction at 105 °C. (b) Selected 24 difucosylated nona- and decasaccharide fractions were repurified using the same column at either 105 or 145 °C. Fraction numbers in blue: difucosylated HMOs with octaose backbone; fraction numbers in red: novel structures.

Based on the assessment of PGC-HPLC conditions shown above, UHT was used for the third dimension PGC-HPLC for subfractionation of the “DP10” fraction. High-resolution separation was achieved at 105 °C, and a total of 70 fractions were obtained (Figure 1a). Negative-ion ESI-MS analysis (Table S1) showed that these were mainly the HMOs containing octaose backbone with 2–4 Fuc residues (e.g., fractions #9 to #54) and decaose backbone with 0–3 Fuc (e.g., #45 to #70), in addition to some minor fractions with hexaose (#1 to #8) and heptaose backbones (#18).

Selected 24 difucosylated decasaccharide fractions were repurified using the same column at either 105 or 145 °C (Figure 1b). A total of 16 difucosylated decasaccharides on different octaose backbones and a difucosylated nonasaccharide on a heptaose backbone were selected for detailed sequence analysis after purity assessment using analytical PGC-HPLC at 105 °C (Figure S6).

### Sequence Determination of Difucosylated Decasaccharides with Linear and Branched Octaose Backbones by Negative-Ion ESI-MS^n^

The strategy for determination of linear^16^ and branched^17^ backbone sequences and Lewis epitopes^16,30^ using negative-ion ESI-MS^n^ is illustrated in Figure S7.

Fraction #21a and #21b can be readily deduced as linear sequences DF-*para*-LNO I (Figure S8a) and DF-*para*-LNnO I (Figure S8b), respectively, from the complete sets of glycosidic C-type ions C_1_ to C_7_. The locations of the two Fuc residues were determined by the 146 Da increment (203 + 146 = 349 Da) between the respective adjacent C-ions (e.g., between C_4_ and C_3_: 893–544 = 349, and between C_6_ and C_5_: 1404–1055 = 349 Da).^16^ The Fuc 3-linkage to the internal −GlcNAc1–3 of Le^x^ can be assigned by the double cleavage D ions (D_4–3β_*m*/*z* 729 and D_6–5β_*m*/*z* 1240), which is characteristic for 3-linaked residues.^16^ The nonreducing terminal Type 1 and Type 2 chains with Gal1–3GlcNAc1– and Gal1–4GclNAc1– sequences, respectively, can be determined by the D_2–1_ ion at *m*/*z* 202 for the 3-linked GlcNAc (Figure S8a) and the ^0,2^A_2_ doublet *m*/*z* 263/281 (Figure S8b).^16^ The reducing terminal −4Glc can be assigned by the doubly charged ions ^2,4^A_8_ and ^0,2^A_8_-h at *m*/*z* 803.7 and 824.8, respectively. Thus, the two linear decasaccharides DF-*para*-LNO I and DF-*para*-LNnO I were both obtained with two internal Le^x^ (*in*Le^x^) (Table S2). The former has not been reported previously.

For decasaccharides with a conventional branched octaose backbone, product-ion scanning using the doubly charged molecular ion [M-2H]^2–^ (*m*/*z* 863.8) gave clear information on the branching point at the Gal of the reducing terminal lactose by D_5–4β_ (*m*/*z* 1037), e.g. in the case of #24b (Figure S9a). As established previously,^17^ sequence information on both 3- and 6-branches can be obtained from the product-ion spectrum of [M-2H]^2–^. D_4β-3β_*m*/*z* 364 and ^0,2^A_2α_ doublet *m*/*z* 281/263 indicate a nonreducing terminal Le^x^ and type 2 chain (Gal1–4GlcNAc−),^16^ respectively. Further MS^3^ scanning using the branching ion D_5–4β_ (*m*/*z* 1037) produced fragment ions only from the 6-branch (Figure S9b), in the product-ion spectrum of which the *m*/*z* 281/263 doublet unambiguously identified the type 2 chain on the 6-branch, and therefore, the Le^x^ epitope shown in the spectrum of [M-2H]^2–^ should be on the 3-branch. The internal Le^x^ ion (D_4α-3α′_*m*/*z* 729) (Figure S8a) is also on the 6-branch. Other difucosylated decasaccharide isomers with conventional branched octaose backbones were similarly identified (Table S2).

### Determination of Novel Difucosyated Branched Octa- and Heptaose Backbones by Negative-Ion ESI-MS^n^

**Figure 2:**
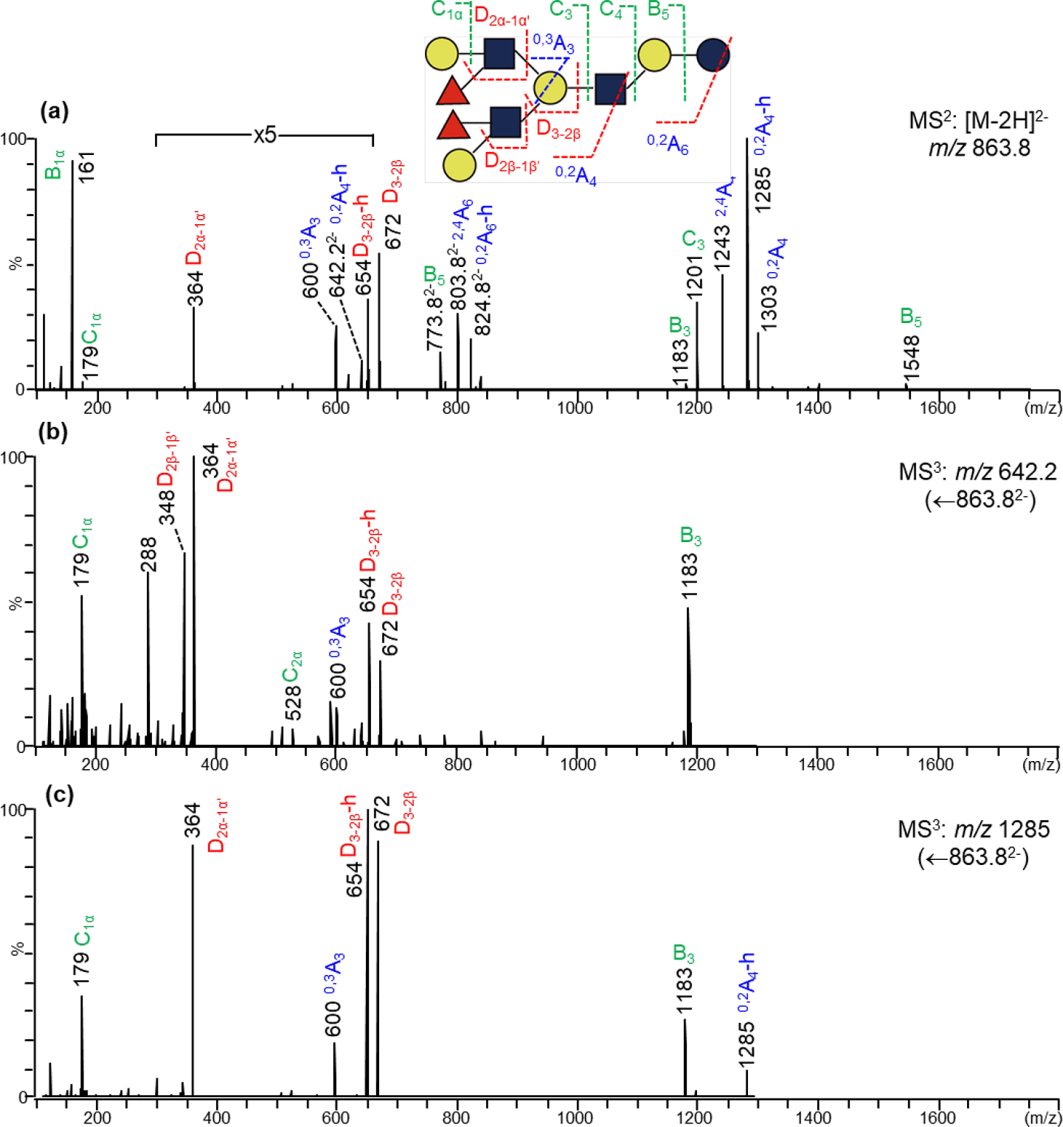
Negative-ion MS^n^ analysis of fraction #44c (DF-*novo*-LNO I). (a) MS^2^ of [M-2H]^2–^*m*/*z* 863.8; (b) MS^3^ of [^0,2^A_4_-h]^2–^*m*/*z* 642.2; (c) MS^3^ of ^0,2^A_4_-h *m*/*z* 1285.

The novel backbone sequence of #44c can be assigned by the branching fragment ion D_3–2β_ at *m*/*z* = 672 (Figure 2a). However, the feature of both 3- and 6-branched sequences from product-ion scanning of [M-2H]^2–^ is absent. Only the 6-branched sequence containing a single Fuc bearing the Le^x^ epitope with an ion D_2α-1α_*m*/*z* 364 was observed. This is because the branching point is farther from the reducing terminus. For this novel octaose backbone, the branching point is at the second Gal residue from the reducing end, i.e., the internal Gal rather than the lactose Gal. As only the doubly charged ion from a reducing side Glc/GlcNAc residue next to the branching point can produce both 3- and 6-branch information, selection of a doubly charged ion from the reducing side GlcNAc is important. Unfortunately, although some intense singly charged ions from this GlcNAc were present in the MS^2^ spectrum, only a very weak doubly charged ^0,2^A_4_-h ion (*m*/*z* 642.2) can be found (Figure 2a) and used as the precursor for further product-ion scanning. Although very weak, the MS^3^ spectrum (Figure 2b) obtained from it still gave clear sequence information on both 3- and 6- branches. It is apparent that both Le^a^ (D-ion *m*/*z* 348) and Le^x^ (D-ion *m*/*z* 364) epitopes are present in fraction #44c. Similar to the conventional octaose branch backbones, further MS^3^ scanning using the singly charged branching ion ^0,2^A_4_-h ion (*m*/*z* 1285) as the precursor (Figure 2c) gave a spectrum showing that Le^x^-specific ion (*m*/*z* 364) at the 6-branch, and therefore, the Le^a^ epitope (with *m*/*z* 348) should be at the 3-branch. Thus, a difucosylated decasaccharide on a novel branched octaose backbone with a Le^a^ and a Le^x^ terminal sequence can be proposed for fraction #44c (Figure 2 and Table S2).

**Figure 3:**
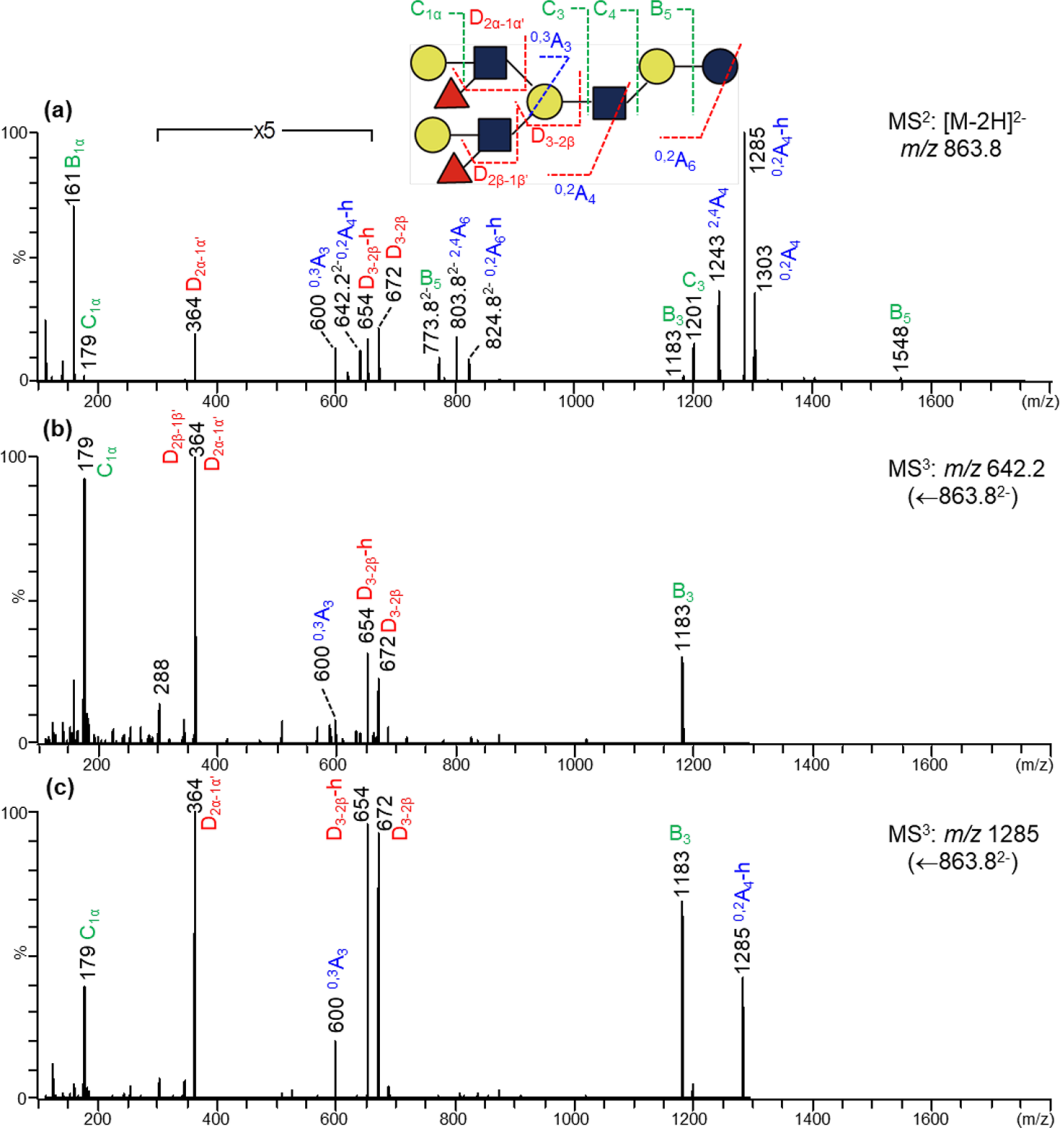
Negative-ion MS^n^ analysis of fraction #42b (DF-*novo*-LNnO I). (a) MS^2^ of [M-2H]^2–^*m*/*z* 863.8; (b) MS^3^ of [^0,2^A_4_-h]^2–^*m*/*z* 642.2; (c) MS^3^ of ^0,2^A_4_-h *m*/*z* 1285.

Fraction #42b with the same novel octaose backbone (Figure 3) can be deduced in a similar way. However, in this case, both branches contain the same Le^x^ epitopes as only the Le^x^-specific ion *m*/*z* 364 was present in both product-ion spectra of the doubly charged ^0,2^A_4_-h ion (*m*/*z* 642.2) (Figure 3b) and the singly charged ^0,2^A_4_-h ion (*m*/*z* 1285) (Figure 3c). An additional two difucosylated HMOs on the *novo*-octaose backbone were proposed for fractions #52b with a blood group H and a Le^x^ at the 3- and 6-branch, respectively (Figure S10 and Table S2), and #46d with a Le^y^ at the 3-branch (Figure S11 and Table S2).

**Figure 4:**
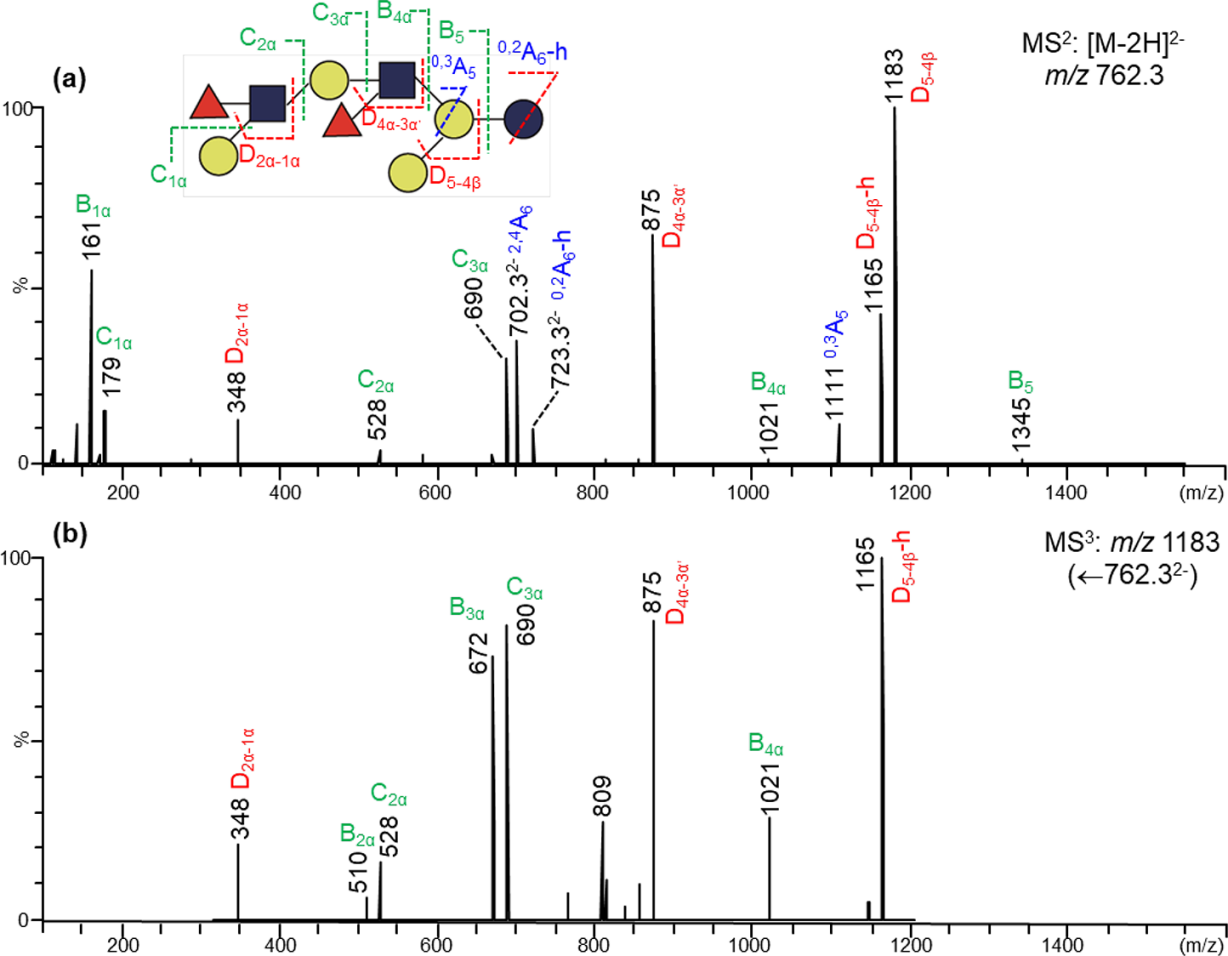
Negative-ion MS^n^ of fraction #18a (DF*-novo-*Hepta). (a) MS^2^ of [M-2H]^2–^*m*/*z* 762.3; (b) MS^3^ of D_5–4β_*m*/*z* 1183.

Fraction #18a gave a molecular mass of 1525.5 with a composition of Fuc2, Hex5, and GlcNAc2 (Table S1), indicating a potentially interesting and unusual heptaose backbone. The product-ion spectrum from the doubly charged [M-2H]^2–^ (Figure 4a) clearly showed a Gal as a single monosaccharide residue at the 3-branch by the double cleavage of D_5–4β_ and its dehydrate ion at *m*/*z* 1183 and 1165, respectively. Further demonstration of the 6-linked branch can be obtained by MS^3^ of D_5–4β_*m*/*z* 1183 (Figure 4b). A terminal Le^a^ epitope and an internal Le^x^ epitope were assigned by the two D-type ions at *m*/*z* 348 and 875 (Figure 4b and Table S2), respectively.

### Validation of Structural Assignment by NMR Spectroscopy

**Figure 5:**
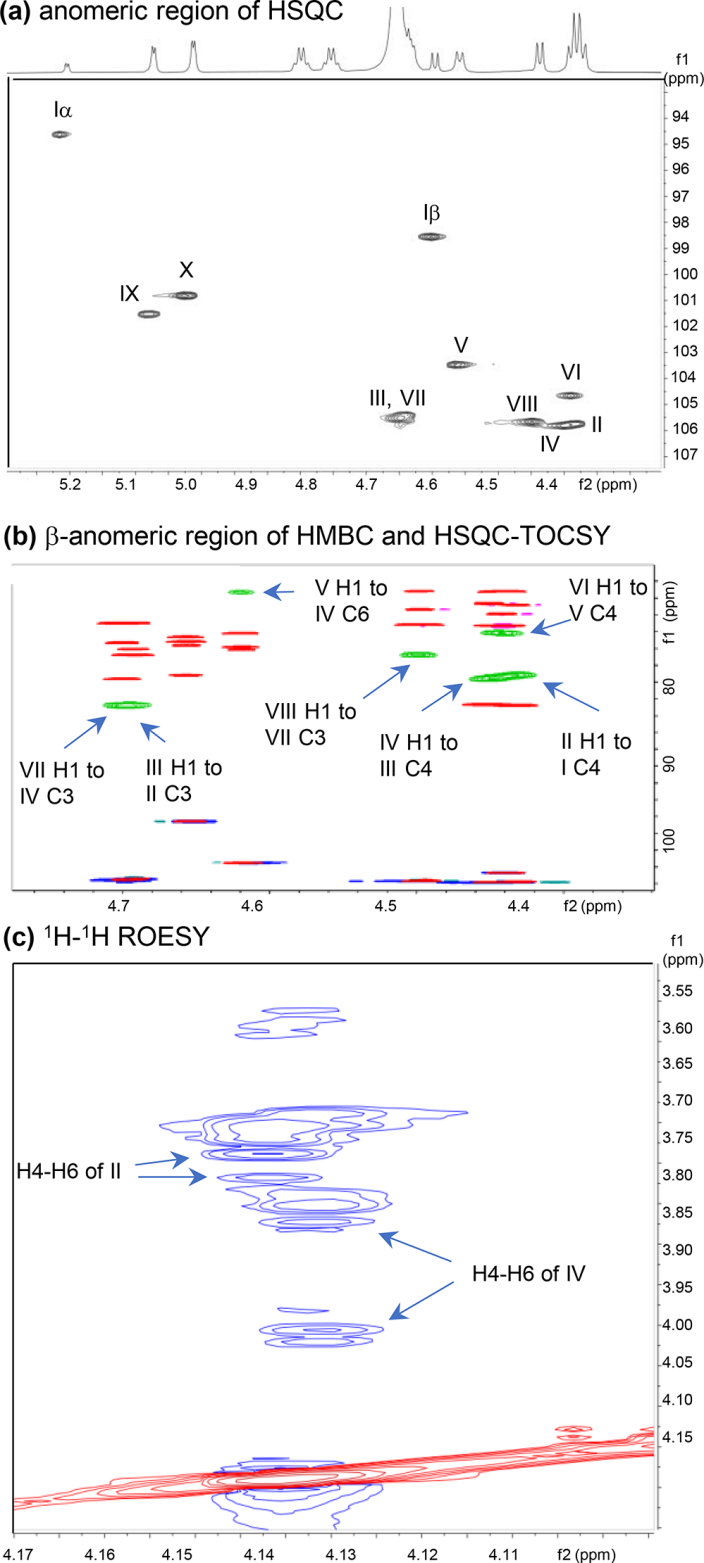
NMR spectra of fraction #44c (DF-*novo*-LNO I). (a) The anomeric region (^1^H 5.3 ppm to 4.3 ppm) of the HSQC spectrum. (b) Expansion of the β-anomeric region (^1^H 4.8 ppm to 4.3 ppm) of the HSQC (blue) and HSQC-TOCSY (red) spectra, overlaid with the HMBC spectrum (green), to define sequence and linkage of monosaccharide residues. (c) Expansion of the ^1^H–^1^H ROESY spectrum for the differential assignment of residues II and IV.

NMR spectra for DF-*novo*-LNO I (fraction #44c) and DF-*novo*-LNnO I (fraction #42b) were recorded at 950 MHz and assigned using 2D heteronuclear ^1^H/^13^C NMR spectra. The anomeric region of the HSQC spectrum of DF-*novo*-LNO I is shown in Figure 5a. Heteronuclear spectroscopy can discriminate between signals that are overcrowded in the ^1^H dimension. Figure 5b shows an expansion of the β-anomeric region of the HSQC (blue) and HSQC-TOCSY (red) spectra of DF-*novo*-LNO I, overlaid with the HMBC spectrum (green). The HMBC peaks illustrated are inter-residue cross-peaks between H1 of one residue and the carbon immediately across the glycosidic linkage, defining both sequence and linkage positions. ^1^H and ^13^C chemical shifts for DF-*novo*-LNO I are summarized in Table S3a (GlcNAc, Glc, and Gal) and Table S3b (Fuc).

In order to establish whether the 6-branch is attached to the backbone at residue Gal II or Gal IV, further reasoning and experimental work were necessary. As is common for Gal residues, the HSQC-TOCSY spectrum can be traced securely only between C1/H1 and C4/H4 for both Gal II and Gal IV. The two spectra are similar; both residues are substituted at the 3-position, and one of them is also substituted at the 6-position.

H1 of GlcNAc V is linked by an inter-residue HMBC connection to glycosylated C6 of Gal at 71.2 ppm (H, H′ 3.96, 3.82) (Figure 5b). H4 of II (4.142 ppm) and H4 of IV (4.135 ppm) are overlapped but are distinguishable. A faint HSQC-TOCSY cross-peak (not illustrated) links H4 of II to C6 at 63.7 ppm (H6, H6′ 3.78, 3.74 ppm), consistent with a nonsubstituted C6.

Further evidence was sought from 2D ROESY spectroscopy. The spectrum obtained (Figure 5c) showed clear ROESY cross-peaks from H4 of II to H6,6′ at 3.78 and 3.74 ppm, and from H4 of IV to H6 at 3.96 and 3.82 ppm, confirming the position of the 6-branch at residue IV.

Two fucose spin systems are traceable through HSQC-TOCSY, H2BC, and HMBC spectra, as described in Supplementary Results.

NMR assignments for DF-*novo*-LNnO I (fraction #42b) were obtained in the same manner, as illustrated in Figure S12, Table S3c,d, and Supplementary Results.

The relatively small amount of sample #18a available was insufficient for heteronuclear NMR spectroscopy except for HSQC, but ^1^H homonuclear 2-dimensional TOCSY and ROESY spectra gave almost complete ^1^H assignments and clear inter-residue ROESY connectivity corroborating the structures indicated by MS^3^ (Figure 4). Assignments for each monosaccharide residue are summarized in Table S4. Detailed ROESY interpretation is shown in the Supplementary Results; the Galβ1–3Gal motif is strongly supported by the two well-resolved inter-residue ROE cross peaks from Gal VI H1 to Gal II H3 and H4 (Figure S13).

### Oligosaccharide Microarray Analysis of the Peripheral Epitopes

To validate the assignment of the epitopes, the 17 purified HMOs together with 14 controls selected from HMOs were converted to fluorescent BABI-probes for the construction of covalent microarrays on NHS-functionalized glass slides. The resulting microarrays were probed with 3 plant lectins (AAL, ECL, and UEA I) and 8 anticarbohydrate antibodies (antibodies against Le^a^, Le^b^, Le^x^, Le^y^, H-T1, H-T2, blood-group A, and blood-group B) with well-defined carbohydrate binding specificities (Table 1 and Figure S14; background color schemes in both the table and figures highlight the structural features and blood-group ABH and Lewis epitopes of the HMO probes and controls). As expected, AAL showed binding signals to all Fuc-containing oligosaccharides (Figure S14a), whereas UEA I (Figure S14b), anti-H-T2 (Figure S14c), and anti-Le^y^ (Figure S14d) did not show any binding signals as H-type 2 and Le^y^ motifs are not present in any of the HMOs identified in the isolated fractions or in any of the controls selected. ECL only recognized HMOs with terminal type 2 chains (Figure S14e). The anti-Le^a^ exhibited specific binding signal to all HMOs with Le^a^ epitope (Figure S14f), while anti-Le^b^ recognized HMOs with Le^b^ epitope (Figure S14g), and not unexpectedly, anti-Le^b^ also showed weak binding signals to Le^a^-containing probes, consistent with previous knowledge.^26^ The anti-H type 1 (Figure S14h), antiblood-group A and antiblood-group B signals (Figure S14i,j) were all as expected.

**Table 1:**
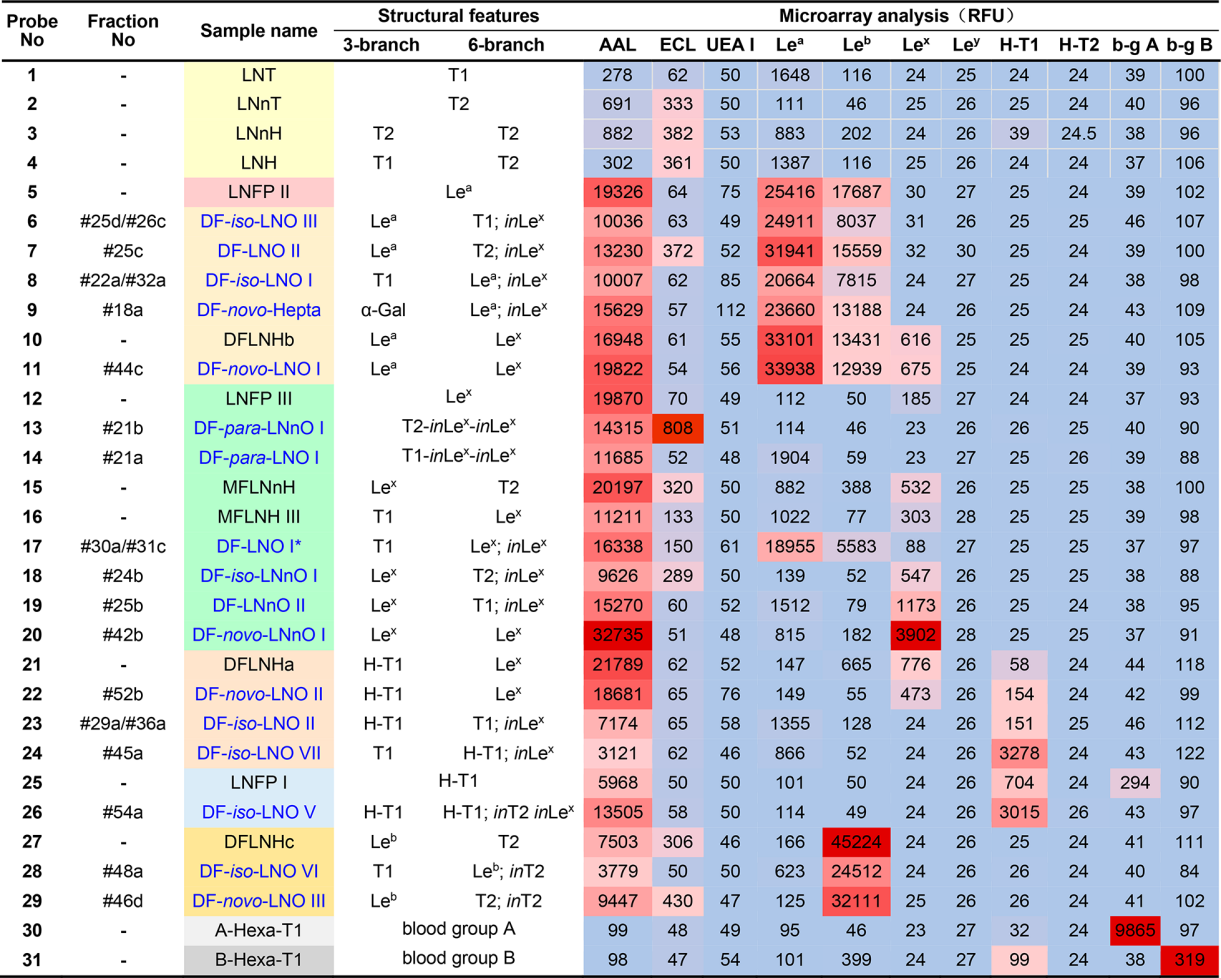
Microarray Analysis of the Difucosylated HMOs with Heptaose and Octaose Backbones^a^

a T1, type 1 chain; T2, type 2 chain; *in*, internal Lewis (Le) epitope; b-g: blood group; samples in black font are included as controls; *purity <80%. Sample names in blue are described in the present study, and those in black are HMO controls. The color shades represent different features of the HMOs (see Figure S13 for details). HMO probes were printed in two concentrations: 25 μM and 50 μM, and 50 μM was used for the heatmap presentation, in which dark red indicates highest binding intensity and light blue indicates weakest or no binding signal.

In this study, the activity of anti-Le^x^ antibody is relatively weak, and for most Le^x^-containing probes including the control LNFP III (probe 12) the fluorescent intensities were below 1,000 counts (Table 1 and Figure S14k). Only DF-*novo*-LNnO I (probe 20, fraction #42b) with double terminal Le^x^ epitopes showed a good binding signal. It is interesting to note that the internal Le^x^ epitope showed no binding signal, e.g., in the case of probes 23 and 24, in agreement with our recent results obtained from microarray binding analysis (Tajadura-Ortega, Chai and colleagues, unpublished) of a comprehensive panel of synthetic sequences containing Lewis antigens at different terminal and internal positions of the backbones.^31^ DF-LNO I (probe 17, fraction #30a/#31c) showed very weak ECL and Le^x^ binding signals but also exhibited some Le^a^ signal as this fraction is a mixture containing ∼40% impurity, among which DF-LNO II with Le^a^ and T2 epitopes was the major one. This is consistent with ESI-MS^4^ (not shown) and purity analysis (Figure S6).

The microarray binding results of all 17 selected HMOs are consistent with ESI-MS^n^ and NMR analyses in terms of epitope assignments.

## Conclusions

Defining the fine structures of oligosaccharides is extremely important for understanding the structural basis of their diverse functions. Unlike N-glycans, HMOs and mucin type O-glycans are not only functionally but also structurally similar^32^ and can have many different backbones and multiple fucose residues forming different isomeric recognition motifs, posing considerable challenge to their separation and structural characterization.

UHT preparative PGC-HPLC, as demonstrated here, can overcome the α/β splitting problem due to the rapid switching between the α and β anomeric forms leading to peak splitting collapses and thus provides a high-efficiency and high-resolution method for separation of reducing glycans obtained from natural glycome sources. As the high temperature used in this study is much lower than the critical temperature of the supercritical fluid ACN/H_2_O, this ensures the solvent is in the liquid state during sample elution. Although the temperature used is high, the column back pressure is reduced due to the reduced solvent viscosity.

For the neutral oligosaccharides, negative-ion ESI-MS/MS is important for the analysis of linear and branched sequences. The combined use of singly and doubly charged molecular deprotonated molecular ions has been successfully used in assignment of many novel sequences of HMOs on branched backbones. However, for the more complex HMOs with DP ≥ 10 and novel branched backbones, MS/MS may not be sufficient, and MS^n^ is required. For example, for MS^3^ scanning the dehydrated ^0,2^A-h ion of the GlcNAc residue at the reducing side of the branching Gal is selected as the unique precursor. As a result, from the highly complex HMO fraction, at least 70 subfractions were detected, and 16 isomeric difucosylated decasaccharides on different octaose backbones and one difucosylated nonasaccharide on a heptaose backbone were isolated, purified, and characterized, among which six are novel sequences. In this and several previous studies,^16,17,25,30^ negative-ion ESI-CID-MS^n^ has played a key role in sequencing of HMOs and other reducing sugars.^18^ However, for complete structural assignment, NMR is always important when a sufficient amount of material is available, as demonstrated here. The sources and the synthetic pathways of oligosaccharides are also important knowledge in the determination of their structures.

The *novo*-octaose backbone and its monofucosylated analogue as AEAB derivatives have been described previously by MS analysis only.^26^ The latter was assigned in a complex mixture of three isomeric structures by product-ion scanning using exactly the same molecular ion precursor. The detection and assignment of the odd-numbered *novo*-heptaose backbone was unexpected as it has not been found in milk, although tetraose backbone from marsupial milk^33^ and pentaose backbone from human milk^34^ with Galβ1–3Gal linkage have been described.

The results presented here can serve to demonstrate further the complexity of HMOs. The method developed in this work can be used for high-efficiency separation and high-sensitivity sequence analysis of large-sized and complex reducing oligosaccharides with multiple isomeric sequences obtained from other glycome sources, e.g., the very challenging mucin O-glycomes when released in nonreducing conditions.

## Supporting Information Available

Additional information on results and methods; PGC-HPLC, SI-MS^n^, and NMR results (Tables S1–S4); HPLC system and additional optimimization, chromatography, negative-ion MS^n^, and NMR results (Figures S1–S14) (PDF)
