# Supplementary material for: Isolation of Human Milk Difucosyl Nona- and Decasaccharides by Ultrahigh-Temperature Preparative PGC-HPLC and Identification of Novel Difucosylated Heptaose and Octaose Backbones by Negative-Ion ESI-MSn
Source: Anal Chem. 2024 Apr 15;96(16):6170–9. doi: 10.1021/acs.analchem.3c05008 (PMC11044106; doi:10.1021/acs.analchem.3c05008)
Supplement: Supplementary file 1 — ac3c05008_si_001.pdf [file ac3c05008_si_001.pdf]

SUPPORTING INFORMATION

Isolation of Human Milk Difucosyl Nona- and Decasaccharides by Ultrahigh Temperature Preparative PGC-HPLC and Identification of Novel Difucosylated Heptaose and Octaose Backbones by Negative-Ion ESI-MS^n

Cuiyan Cao,[a†] Yiming Cheng,[b‡] Yi Zheng,[a†] Beibei Huang,[b‡] Zhimou Guo,[a†,c§] Long Yu,[a†,c§] Barbara Mulloy,[d∥] Virginia Tajadura-Ortega,[d∥] Wengang Chai,[d∥,*] Jingyu Yan,[a†,c§,*] Xinmiao Liang[a†,c§,*]

[a]Dalian Institute of Chemical Physics, Chinese Academy of Sciences, Key Laboratory of Separation Science for Analytical Chemistry, Dalian 116023, China

[b]Jiangxi Provincial Key Laboratory for Pharmacodynamic Material Basis of Traditional Chinese Medicine, Ganjiang Chinese Medicine Innovation Center, Nanchang 330000, China

[c]University of Chinese Academy of Sciences, Beijing 100049, China

[d]Glycosciences Laboratory, Faculty of Medicine, Imperial College London, Hammersmith Campus, London W12 0NN, United Kingdom

*Correspondence to: yanjingyu@dicp.ac.cn; w.chai@imperial.ac.uk ; liangxm@dicp.ac.cn

CONTENTS

Supplementary Results

Supplementary Methods

Table S1. Main composition information of 70 HPLC fractions from PGC-HPLC at 105 ^{o}C.

Table S2. Identification of difucosylated HMOs with heptaose and octaose backbones by ESI-MS^{n}.

Table S3. NMR assignment of DF-*novo*-LNO I (fraction #44c) and DF-*novo*-LNnO I (fraction #42b).

Table S4. NMR assignment of DF-*novo*-Hepta (fraction #18a).

Figure S1. Ultrahigh temperature HPLC system.

Figure S2. Optimization of PGC-HPLC conditions using a standard mixture of LNFP I, II and III.

Figure S3. Optimization of temperature for PGC-HPLC separation of DF-*novo*-LNnO II and DF-*novo*-LNO I.

Figure S4. Stabilities of sialylated and neutral HMOs under different ultrahigh temperature conditions.

Figure S5. Group separation of HMOs based on two-dimensional hydrophilic chromatography.

Figure S6. Purity analysis of 17 isolated HMOs by analytical scale PGC-HPLC.

Figure S7. Workflow for ESI-MS^{n} analysis of difucosylated HMO isomers with octaose backbones.

Figure S8. Negative-ion MS^{2} of fraction #21a (DF-*para*-LNO I) and fraction #21b (DF-*para*-LNnO I).

Figure S9. Negative-ion MS^{n} of fraction #24b (DF-*iso*-LNnO I).

Figure S10. Negative-ion MS^{n} of fraction #52b (DF-*novo*-LNO II).

Figure S11. Negative-ion MS^{n} analysis of fraction #46d (DF-*novo*-LNO III).

Figure S12. NMR spectra of DF-*novo*-LNnO I.

Figure S13. Overlaid TOCSY (blue) and ROESY (pink) NMR spectra of fraction #18a (DF-*novo*-Hepta).

Figure S14. Microarray binding of AAL (a), UEA I (b), anti- H type 2(c), anti-Le^{y} (d), ECL (e), anti-Le^{a} (f), anti-Le^{b} (g), anti-H type 1 (h), anti-blood group A (i), anti-blood group B (j), and anti-Le^{x} (k).

SUPPLEMENTARY RESULTS

Complete structural assignment of novel difucosylated HMOs on branched octaose backbone by NMR

NMR spectra for the decasacccharides DF-*novo*-LNO I (fraction #44c) and DF-*novo*-LNnO I (fraction #42b) were recorded at 950 MHz in D_2O, and assigned using 2D heteronuclear $^1H/^{13}C$ NMR spectra. The anomeric region of the HSQC spectrum of DF-*novo*-LNO I is shown in **Figure 5a**. Heteronuclear spectroscopy can discriminate between signals that are overcrowded in the ^1H dimension, such as the β-anomeric region between 4.35 and 4.75 ppm, though some pairs of signals still overlap, for example the anomeric cross-peaks from GlcNAc residues III and VII, and residues IV and II (**Figure 5a**).

HSQC-TOCSY and H2BC spectra were used to assign the remaining signals from GlcNAc and Glc residues, and Gal H2, H3 and H4 signals. The small coupling constant value between H4 and H5 of Gal (and Fuc) residues means that the HSQC-TOCSY H4/C4-H5/C5 cross-peaks are faint or missing.

Sequence determination and completion of fucose residue assignment relies on long-range HMBC cross-peaks. **Figure 5b** shows an expansion of the β-anomeric region of the HSQC (blue) and HSQC-TOCSY (red) spectra of DF-*novo*-LNO I, overlaid with the HMBC spectrum (green). The HMBC peaks illustrated are inter-residue cross-peaks between H1 of one residue and the carbon immediately across the glycosidic linkage, defining both sequence and linkage positions. ^1H and ^{13}C chemical shifts for DF-*novo*-LNO I are summarized in **Table S3a** (GlcNAc. Glc and Gal) and **Table S3b** (Fuc).

In order to establish whether the 6-branch is attached to the backbone at residue Gal II or residue Gal IV, further reasoning and experimental work was necessary. As is common for Gal residues, the HSQC-TOCSY spectrum can be traced securely only between C1/H1 and C4/H4 for both Gal II and Gal IV. The two spectra are very similar; both residues are substituted at the 3- position and one of them is also substituted at the 6-position.

H1 of GlcNAc V is linked by an inter-residue HMBC connection to glycosylated C6 of Gal at 71.2 ppm (H, H' 3.96, 3.82) (**Figure 5b**). H4 of II (4.142 ppm) and H4 of IV (4.135 ppm) are overlapped but distinguishable. A faint HSQC-TOCSY cross peak (not illustrated) links H4 of II to C6 at 63.7 ppm (H6, H6' 3.78, 3.74 ppm), consistent with a non-substituted C6.

Further evidence was sought from 2D ROESY spectroscopy. The spectrum obtained (**Figure 5c**) showed clear ROESY cross-peaks from H4 of II to H6,6' at 3.78 and 3.74 ppm, and from H4 of IV to H6 at 3.96 and 3.82 ppm, confirming the position of the 6-branch at residue IV.

Two fucose spin systems are traceable through HSQC-TOCSY, H2BC and HMBC spectra, identified by the characteristic H6/C6 methyl signals. Differential assignment of the two spin systems to residues IX and X is made difficult by the absence of inter-residue HMBC peaks, so assignments to IX and X are based on close similarity between current results and literature values, as shown in **Table S3b**.

NMR assignments for DF-*novo*-LNnO I were obtained in the same manner, as illustrated in **Figure S12** For this compound heavily overlapped signals (particularly in the 1H dimension) from Gal residues VIII VI, IV and II seriously hindered determination of the position of the 6-branch without ambiguity.

^{1}H and ^{13}C chemical shifts for DF-novo-LNnO I are summarised in **Table S3c** (GlcNAc, Glc and Gal) and **Table S3d** (Fuc).

Sequence and linkage validation by NMR of the difucosylated nonasaccharide on a novel heptaose backbone

The relatively small amount of fraction #18a available was insufficient for most heteronuclear NMR spectroscopy except for HSQC, but ^{1}H homonuclear 2-dimensional TOCSY and ROESY spectra gave partial assignments and clear inter-residue ROESY connectivity (**Figure S13**) corroborating the structures indicated by mass spectrometric methods. Assignments for each monosaccharide residue, as summarized in **Tables S4**, are based on a

combination of experimental data (TOCSY, ROESY cross-peaks) and comparison with NMR data for similar monosaccharide residues in DF-*novo*-LNO I and DF-*novo*-LNnO I as described above, as well as literature values for the fucose residues as indicated in the **Table S4**.

ROESY connectivity traced glycosidic linkages through the molecule as follows: VII H1 (4.51 ppm) to V H3 (4.06 ppm), VIII H1 (5.02 ppm) to V H4 (3.76 ppm), V H1 (4.68 ppm) to IV H3 (3.70 ppm), IV H1 (4.43 ppm) to III H4 (3.92 ppm), IX H1 (5.09 ppm) to III H3 (3.88 ppm, coincident with IX H3), III H1 (4.64 ppm) to II H6 (3.85 ppm), VI H1(4.60 ppm) to II H3 (3.82 ppm) and II H4 (4.16 ppm), and II H1 (4.49 ppm) to I H4 (3.63 ppm).

ROESY connectivity listed above is completely compatible with the structure proposed. Other ROESY cross-peaks could tentatively be assigned to H1-H5 connections for Gal residues or could originate in interactions between closely packed residues in the branched structure. H6 and H6' of the 6-substituted Gal II were assigned by ROE cross-peaks from H4 and confirmed by the presence in the HSQC spectrum of cross-peaks to C6 at 71.2 ppm, shifted well downfield of the other H6-C6 cross-peaks. The Galβ1-3Gal motif is strongly supported by the two well-resolved inter-residue ROE cross peaks from Gal VI H1 to Gal II H3 and H4.

SUPPLEMENTARY METHODS

Preparation of fucosylated decasaccharide fraction from human milk. The stored frozen milk sample (~20 L) from different donors was thawed at 4 °C and filtered sequentially through two hollow fiber membranes (750 and 50 kDa, Shandong Bona Biology, Shandong, China) to remove the high molecular mass proteins and lipids aggregates. The collected filtrate was mixed with an equal volume of ethanol to prepare the final HMO solution at 100 mg/mL. The preparative work was performed on a Hanbon preparative liquid chromatograph system (Hanbang Sci & Tech, Huai'an, China) equipped with Hanbon-NP7000C binary gradient pump, Hanbon-NU3000C UV-visible dual wavelength detector, manual sampler and Easychrom-1000 chromatographic workstation.

First dimension preparative HPLC on a Click TE-GSH column (100 × 250 mm, 5 μm, Acchrom, Wenling, China) was used to remove the large amount of lactose and separate the neutral from sialylated oligosaccharides by the solvent system of EtOH/H_2O at a flow rate of 240 mL/min and detection by UV at 195nm. The injection volume was 400 mL (100 mg/mL) and the elution solvent gradient was: 0-20 min, isocratic at 80/20 (EtOH/H_2O, v/v); 20-55 min, 80/20 to 40/60 (EtOH/H_2O, v/v). The pooled fraction containing neutral oligosaccharides with the highest DPs (DP ≥ 10, F5) was collected for further fractionation.

The second dimension preparative HPLC was carried out on a HILIC amide column (100 × 250 mm, 10 μm, Acchrom) for fractionation of the neutral high molecular mass HMOs (F5). The injection volume was 80 mL (50 mg/mL) and elution solvent was CH_3CN/H_2O using a gradient of 65/35 to 30/70 (CH_3CN/H_2O, v/v) in 60 min at a flow rate of 80 mL/min and detection by UV 195 nm. The manually collected fraction "DP10" was subjected to final fractionation.

The third dimension HPLC was performed on the purpose-built ultrahigh temperature HPLC system and a Hypercarb PGC column (Thermo Fisher Scientific). Separation of "DP10" fraction was carried out at 105 ℃ on a PGC column (4.6 ×150 mm, 5 μm) with the following solvent gradient: 0-120 min, 10/90 to 25/75 (CH_3CN/H_2O); 120-180 min, 25/75 to 50/50 (CH_3CN/H_2O), at a flow rate of 1.0 mL/min and detection by UV 195 nm. The injection volume was 20 μL at a concentration of 250 mg/mL and 70 fractions were manually collected, and concentrated and dried by lyophilization. The 24 main fractions (highlighted in blue and red font, **Figure 1b**) containing difucosylated nona- and decasaccharides were further purified on either the same column at 105 ℃ or on a Hypercarb PGC HT column (3.0 × 100 mm, 3 μm, Thermo Fisher Scientific) at 145 ^{o}C with optimized CH_3CN/H_2O gradient (with or without 0.1% formic acid).

HMO microarrays. All the carbohydrate-binding proteins were purchased from commercial sources as listed below. *Aleuria aurantia* lectin (AAL), *Erythrina cristagalli* lectin

(ECL), *Ulex europaeus* agglutinin I (UEA I) were purchased from Vector Laboratories (San Francisco, CA), and antibodies including mouse anti-blood group A (SAB4700674), mouse anti-blood group B, mouse anti-Le^a, mouse anti-Le^b, biotinylated goat anti-mouse IgG and BSA were obtained from Sigma-Aldrich (Saint Louis, MO), and mouse anti-Le^x (P12) and anti-Le^y were from Santa Cruz Biotechnology (Santa Cruz, TX). Goat anti-mouse IgM was from Abmart (Shanghai, China), and streptavidin AF647 (S21374) was from ThermoFisherScientific (Rockford, IL).

The HMO probes were prepared by reductive amination with a fluorescent amino-terminating bifunctional linker BABI (Yan, Zheng and colleagues, unpublished) and printed in quadruplicate on the NHS-functionalized glass slides (Schott Nexterion H, Germany) using sciFLEXARRAYER S3 (SCIENION, Berlin, Germany). For binding assay, the HMO probes were arrayed at concentrations of 25 μM and 50 μM. The microarray procedure was as described by Blixt et al. (*Proc. Natl. Acad. Sci. USA* 2004, 101, 17033-8). Briefly, after blocking with 1% BSA (Millipore-Sigma, Burlington) in TBST (TBS with 0.1% Tween) and washing with TBST, biotinylated plant lectins and monoclonal anti-carbohydrate antibodies with various dilutions (AAL, UEA and ECL, 1:1000; anti-B, 1:20; anti-A, 1:200; anti-Le^a, anti-Le^b, anti-Le^y, anti-H type 1, anti-H type 2 and anti-Le^x, 1:100 diluted in the blocking solution) were added and incubated at ambient temperature for 90 min. The binding signals were detected using AlexaFluor-647-labeled streptavidin (1:1000) or the biotinylated anti-Mouse IgG (1:300) with subsequent AlexaFluor-647-labeled streptavidin. Anti-B antibody was detected with anti-mouse IgM AlexaFluor-680 (1:300). The fluorescence binding signals were measured and quantified using the GenePix Pro 7 software (Molecular Devices).

Table S1. Main composition information of 70 HPLC fractions from PGC-HPLC at 105 °C.

HPLC No	Found	Composition	Calc'd Mol Mass	Theor Mol Mass	HPLC No	Found	Composition	Calc'd Mol Mass	Theor Mol Mass
#1	998.4 (-1)<br>1509.6 (-1)	N1H3F2<br>N2H4F3	999.4<br>1510.6	999.3642<br>1510.5543	#36	1728.7 (-1)*	N3H5F2	1729.7	1729.6286
#2	1655.6 (-1)<br>1509.6 (-1)	N2H4F4<br>N2H4F3	1656.6<br>1510.6	1656.6122<br>1510.5543	#37	1728.7 (-1)<br>1009.9 (-2)	N3H5F2<br>N3H5F4	1729.7<br>2021.8	1729.6286<br>2021.7444
#3	1347.5 (-1)	N2H3F3	1348.5	1348.5015	#38	1728.7 (-1)<br>1119.4 (-2)	N3H5F2<br>N4H6F3	1729.7<br>2240.8	1729.6286<br>2240.8187
#4	1509.6 (-1)	N2H4F3	1510.6	1510.5543	#39	1728.7 (-1)<br>1046.4 (-2)	N3H5F2<br>N4H6F2	1729.7<br>2094.8	1729.6286<br>2094.7608
#5	1655.6 (-1)*	N2H4F4	1656.6	1656.6122	#40	1728.7 (-1)<br>1655.6 (-1)	N3H5F2<br>N2H4F4	1729.6<br>1656.6	1729.6286<br>1656.6122
#6	1509.6 (-1)	N2H4F3	1510.6	1510.5543	#41	1728.7 (-1)<br>1582.6 (-1)	N3H5F2<br>N3H5F1	1729.7<br>1583.6	1729.6286<br>1583.5707
#7	1509.6 (-1)	N2H4F3	1510.6	1510.5543	#42	1728.7 (-1)<br>1874.7 (-1)	N3H5F2<br>N3H5F3	1729.7<br>1875.7	1729.6286<br>1875.6865
#8	1509.6 (-1)	N2H4F3	1510.6	1510.5543	#43	1874.7 (-1)<br>1728.7 (-1)	N3H5F3<br>N3H5F2	1875.7<br>1729.7	1875.6865<br>1729.6286
#9	1874.7 (-1)*	N3H5F3	1875.7	1875.6865	#44	1874.7 (-1)<br>1728.7 (-1)	N3H5F3<br>N3H5F2	1875.7<br>1729.7	1875.6865<br>1729.6286
#10	1363.5 (-1)*	N2H4F2	1364.5	1364.4964	#45	1728.7 (-1)<br>1046.4 (-2)	N3H5F2<br>N4H6F2	1729.7<br>2094.8	1729.6286<br>2094.7608
#11	1874.7 (-1)	N3H5F3	1875.7	1875.6865	#46	1728.7 (-1)<br>1874.7 (-1)	N3H5F2<br>N3H5F3	1729.7<br>1875.7	1729.6286<br>1875.6865
#12	1874.7 (-1)*	N3H5F3	1875.7	1875.6865	#47	1728.7 (-1)<br>1046.4 (-2)	N3H5F2<br>N4H6F2	1729.7<br>2094.8	1729.6286<br>2094.7608
#13	1874.7 (-1)*	N3H5F3	1875.7	1875.6865	#48	1728.7 (-1)*	N3H5F2	1729.6	1729.6286
#14	1874.7 (-1)*	N3H5F3	1875.7	1875.6865	#49	1046.4 (-2)<br>1728.7 (-1)	N4H6F2<br>N3H5F2	2094.8<br>1729.7	2094.7608<br>1729.6286
#15	1874.7 (-1)<br>1655.6 (-1)	N3H5F3<br>N2H4F4	1875.7<br>1656.6	1875.6865<br>1656.6122	#50	1046.4 (-2)<br>1728.7 (-1)	N4H6F2<br>N3H5F2	2094.8<br>1729.7	2094.7608<br>1729.6286
#16	1655.6 (-1)<br>1874.7 (-1)	N2H4F4<br>N3H5F3	1656.6<br>1875.7	1656.6122<br>1875.6865	#51	1046.4 (-2)<br>1728.7 (-1)	N4H6F2<br>N3H5F2	2094.8<br>1729.7	2094.7608<br>1729.6286
#17	1874.7 (-1)<br>1509.6 (-1)	N3H5F3<br>N2H4F3	1875.7<br>1510.6	1875.6865<br>1510.5543	#52	1046.4 (-2)<br>1728.7 (-1)	N4H6F2<br>N3H5F2	2094.8<br>1729.7	2094.7608<br>1729.6286
#18	1874.7 (-1)<br>1525.4 (-1)	N3H5F3<br>N2H5F2	1875.7<br>1526.4	1875.6865<br>1526.5492	#53	1046.4 (-2)<br>1947.7 (-1)	N4H6F2<br>N4H6F1	2094.8<br>1948.7	2094.7608<br>1948.7029
#19	1874.7 (-1)<br>1728.6 (-1)	N3H5F3<br>N3H5F2	1875.7<br>1729.7	1875.6865<br>1729.6286	#54	1046.4 (-2)<br>1728.6 (-1)	N4H6F2<br>N3H5F2	2094.8<br>1729.6	2094.7608<br>1729.6286
#20	1874.7 (-1)	N3H5F3	1875.7	1875.6865	#55	1947.7 (-1)*	N4H6F1	1948.7	1948.7029
#21	1728.6 (-1)	N3H5F2*	1729.7	1729.6286	#56	1046.8 (-2)<br>1119.4 (-2)	N4H6F2<br>N4H6F3	2094.8<br>2240.8	2094.7608<br>2240.8187
#22	1874.7 (-1)<br>1728.7 (-1)	N3H5F3<br>N3H5F2	1875.7<br>1729.7	1875.6865<br>1729.6286	#57	1947.7 (-1)*	N4H6F1	1948.7	1948.7029
#23	1874.7 (-1)<br>1728.7 (-1)	N3H5F3<br>N3H5F2	1875.7<br>1729.7	1875.6865<br>1729.6286	#58	1947.7 (-1)*	N4H6F1	1948.7	1948.7029
#24	1874.7 (-1)<br>1728.7 (-1)	N3H5F3<br>N3H5F2	1875.7<br>1729.7	1875.6865<br>1729.6286	#59	1046.4 (-2)*	N4H6F2	2094.8	2094.7608
#25	1874.7 (-1)<br>1728.7 (-1)	N3H5F3<br>N3H5F2	1875.7<br>1729.7	1875.6865<br>1729.6286	#60	1046.4 (-2)<br>1947.7 (-1)	N4H6F2<br>N4H6F1	2094.8<br>1948.7	2094.7608<br>1948.7029

**#26**	1874.7 (-1)<br>1728.7 (-1)	N3H5F3<br>N3H5F2	1875.7<br>1729.6	1875.6865<br>1729.6286	**#61**	1046.4 (-2)<br>1947.7 (-1)	N4H6F2<br>N4H6F1	2094.8<br>1948.7	2094.7608<br>1948.7029
**#27**	1728.7 (-1)<br>1009.8 (-2)	N3H5F2<br>N3H5F4	1729.7<br>2021.6	1729.6286<br>2021.7444	**#62**	1046.4 (-2)<br>1947.7 (-1)	N4H6F2<br>N4H6F1	2094.8<br>1948.7	2094.7608<br>1948.7029
**#28**	1874.7 (-1)<br>1728.7 (-1)	N3H5F3<br>N3H5F2	1875.7<br>1729.7	1875.6865<br>1729.6286	**#63**	1947.7 (-1)*	N4H6F1	1948.7	1948.7029
**#29**	1728.7 (-1)*	N3H5F2	1729.7	1729.6286	**#64**	1947.7 (-1)*	N4H6F1	1948.7	1948.7029
**#30**	1728.7 (-1)<br>1874.7 (-1)	N3H5F2<br>N3H5F3	1729.7<br>1875.7	1729.6286<br>1875.6865	**#65**	1947.7 (-1)*	N4H6F1	1948.7	1948.7029
**#31**	1874.7 (-1)<br>1728.7 (-1)	N3H5F3<br>N3H5F2	1875.7<br>1729.7	1875.6865<br>1729.6286	**#66**	1801.6 (-1)*	N4H6	1802.6	1802.6450
**#32**	1874.7 (-1)<br>1728.7 (-1)	N3H5F2<br>N3H5F3	1729.7<br>1875.7	1729.6286<br>1875.6865	**#67**	1801.6 (-1)*	N4H6	1802.6	1802.6450
**#33**	1009.8 (-2)<br>1728.7 (-1)	N3H5F4<br>N3H5F2	2021.6<br>1729.7	2021.7444<br>1729.6286	**#68**	1801.6 (-1)*	N4H6	1802.6	1802.6450
**#34**	1874.7 (-1)<br>1728.7 (-1)	N3H5F3<br>N3H5F2	1875.7<br>1729.7	1875.6865<br>1729.6286	**#69**	1947.7 (-1)*	N4H6F1	1948.7	1948.7029
**#35**	1874.7 (-1)<br>1728.7 (-1)	N3H5F3<br>N3H5F2	1875.7<br>1729.7	1875.6865<br>1729.6286	**#70**	1801.6 (-1)*	N4H6	1802.6	1802.6450

Note：*refers to other complex composition.

Table S2. Identification of difucosylated HMOs with heptaose and octaose backbones by ESI-MS^{n}.

Fraction No.	Sample name	Structure	Diagnostic fragment ions in negative ion ESI-CID-MS/MS			Amount (mg)	Purity (%)
			backbone	3-branch	6-branch		
**#25c**	DF-LNO II	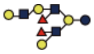	1037/1019	348 (Le^{a})	281/263 (T2); 729 (*in*Le^{x})	0.1	>80
**#30a/#31c**	DF-LNO I*	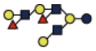	1183/1165	382/202 (T1)	364 (Le^{x}); 875 (*in*Le^{x})	0.5	<60
**#25b**	DF-LNnO II	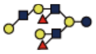	1037/1019	364 (Le^{x})	202 (T1); 729 (*in*Le^{x})	0.2	>90
**#25d/#26c**	DF-*iso*-LNO III	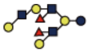	1037/1019	348 (Le^{a})	202 (T1); 729(*in*Le^{x})	0.1	>90
**#29a/#36a**	DF-*iso*-LNO II	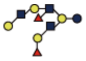	1037/1019	325 (H-T1)	202 (T1); 729 (*in*Le^{x})	0.3	>95
**#54a**	DF-*iso*-LNO V	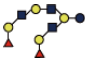	1037/1019	325 (H-T1)	325(H-T1); 792/774 (*in*T2)	0.1	>90
**#22a/#32a**	DF-*iso*-LNO I	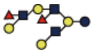	1183/1165	382/202 (T1)	348 (Le^{a}); 875 (*in*Le^{x})	0.2	>80
**#45a**	DF-*iso*-LNO VII	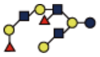	1183/1165	382/202 (T1)	325 (H-T1); 875 (*in*Le^{x})	0.2	>85
**#48a**	DF-*iso*-LNO VI	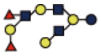	1183/1165	382/202 (T1)	325/348 (Le^{b}); 938/920(*in*T2)	0.6	>90
**#24b**	DF-*iso*-LNnO I	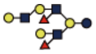	1037/1019	364 (Le^{x})	281/263 (T2) ; 729(*in*Le^{x})	0.1	>95
**#44c**	DF-*novo*-LNO I	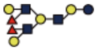	1285/672	348 (Le^{a})	364 (Le^{x})	0.3	>95
**#52b**	DF-*novo*-LNO II	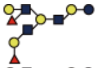	1285/672	325 (H-T1)	364 (Le^{x})	0.2	>85
**#46d**	DF-*novo*-LNO III	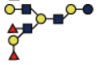	1285/526	325/348 (Le^{b})	281/263 (T2)	0.5	>95
**#42b**	DF-*novo*-LNnO I	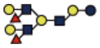	1285/672	364 (Le^{x})	364 (Le^{x})	0.3	>95
**#21a**	DF-*para*-LNO I	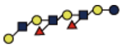	161-382(202)-544-893(729)-1055-1548(1240); T1-*in*Le^{x}-*in*Le^{x}			0.15	>95
**#21b**	DF-*para*-LNnO I	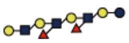	161-382(263/281)-544-893(729)-1055-1548(1240); T2-*in*Le^{x}-*in*Le^{x}			0.10	>95
**#18a**	DF-*novo*-Hepta	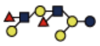	1183/1165	161	348 (Le^{a}); 875 (*in*Le^{x})	0.5	>80

T1, type 1 chain; T2, type 2 chain; *in*, internal Lewis (Le) epitope; fragment ions in blue are diagnostic ions. Oligosaccharides highlighted in red are novel structures.

Table S3. NMR assignment of DF-*novo*-LNO I (fraction #44c) and DF-*novo*-LNnO I (fraction #42b).

(a) ^{1}H and ^{13}C assignments for Glc, GlcNAc and Gal residues of DF-*novo*-LNO I (in ppm)

	VIII<br>**NRGalβ**	VII<br>**GlcNAcβ**	VI<br>**NRGalβ**	V<br>**GlcNAcβ**	IV<br>**Galβ**	III<br>**GlcNAcβ**	II<br>**Galβ**	Ia<br>**RGlcα**	Ib<br>**RGlcβ**
**1**	4.50,105.6	4.69,105.4	4.44,104.6	4.62,103.3	4.45,105.8	4.70,105.4	4.43,105.7	5.22,94.5	4.65,98.4
**2**	3.48,73.2	3.94,58.7	3.49,73.8	3.89,58.4	3.57,72.6	3.81,57.8	3.59,72.7	3.57,73.9	3.28,76.5
**3**	3.61,75.2	**4.07,78.7**	3.64,75.2	3.86,77.7	**3.70,84.7**	3.73,74.9	**3.71,84.6**	3.83,74.1	3.63,77.0
**4**	3.89,71.1	3.75,74.9	3.89,71.1	**3.92,76.1**	4.13,71.1	**3.67,81.6**	4.14,71.1	**3.64,81.2**	**3.64,81.2**
**5**	3.59, 77.7	3.53,78.0	3.56, 77.6	3.58,78.1	3.80,76.3	3.58,77.2	3.72,74.9	3.94,72.9	3.59,77.6
**6**	3.74/3.70, 64.2*	3.9/3.86, 62.4	3.72, 64.4*	3.98/3.84, 62.5	**3.96/3.82, 71.2**	3.98/3.84, 62.6	3.78/3.74, 63.7	ND	3.79/3.94, 62.8

(b) ^{1}H and ^{13}C assignments for Fuc residues of DF-*novo*-LNO I (in ppm)

	**IX** (present study)	**IX** (reference data)	**X** (present study)	**X** (reference data)
**1**	5.08,101.4	5.09	5.02,100.8	5.03
**2**	3.70, 70.5	3.70	3.80,70.6	3.80
**3**	3.89,72.0	3.88	3.88,71.9	3.88
**4**	3.78,74.7	3.78	3.79,74.7	3.79
**5**	4.81,69.5	4.81	4.86,69.6	4.87
**6**	1.16,18.1	1.15	1.16,18.1	1.18

(c) ^{1}H and ^{13}C assignments for Glc, GlcNAc and Gal residues of DF-*novo*-LNnO I (in ppm)

	VIII<br>**NRGalβ**	VII<br>**GlcNAcβ**	VI<br>**NRGalβ**	V<br>**GlcNAcβ**	IV<br>**Galβ**	III<br>**GlcNAcβ**	II<br>**Galβ**	Ia<br>**RGlcα**	Ib<br>**RGlcβ**
**1**	4.46,104.7	4.71,105.2	4.44,104.7	4.62,103.4	4.45,105.6	4.69,105.5	4.43,105.6	5.21,94.6	4.66,98.45
**2**	3.49,74.0	3.95,58.7	3.49,74.0	3.89,58.3	3.56,72.6	3.80,57.9	3.59,72.8	3.57,73.9	3.27,76.5
**3**	3.64,75.2	3.88,77.5	3.64,75.2	3.88,77.6	**3.69,84.6**	3.73,74.9	**3.71,84.7**	3.83,74.1	3.63,77.0
**4**	3.89,71.1	**3.94,75.8**	3.89,71.1	**3.92,76.2**	4.14,71.1	**3.67,81.6**	4.14,71.1	**3.64,81.2**	**3.64,81.1**
**5**	3.60,77.6	3.58, 77.2	3.60, 77.6	3.58,78.1	3.80,76.3	3.60, 77.3	3.70, 77.6	3.94, 72.8	3.59, 77.6
**6**	3.74;3.70, 64.2	3.94;3.83, 62.6	3.74;3.70 64.2	3.97;3.84, 62.5	**3.96;3.81, 71.2**	3.94;3.83, 62.6	3.78;3.74, 63.7	ND	3.94;3.79, 62.8

(d) ^{1}H and ^{13}C assignments for Fuc residues of DF-*novo*-LNnO I (in ppm)

	**IX** (present study)	**IX** (reference data)	**X** (present study)	**X** (reference data)
**1**	5.12,101.3	5.09	5.08,101.5	5.03
**2**	3.69,70.5	3.70	3.69, 70.5	3.80
**3**	3.90,72.0	3.88	3.89,72.0	3.88
**4**	3.79,74.7	3.78	3.79,74.7	3.79
**5**	4.82,69.5	4.81	4.82,69.5	4.87
**6**	1.17,18.1	1.15	1.17,18.1	1.18

Glycosylation positions assigned by HMBC and ROESY cross-peaks in bold; *indicates that assignments may be reversed. Fuc positions in italic. Assignments for all Gal H5,C5 are tentative. ND: not determined.
Reference: Kogelberg et al: *Eur. J. Biochem.* 2004, 271, 1172-1186.

Table S4. NMR assignment of DF-*novo*-Hepta (fraction #18a)

(a) ^{1}H assignments for Glc, GlcNAc and Gal residues of DF-*novo*-Hepta (in ppm)

	VII	VI	V	IV	III	II	Ia	Ib
	**NRGalβ**	**NRGalβ**	**GlcNAcβ**	**Galβ**	**GlcNAcβ**	**Galβ**	**RGlcα**	**RGlcβ**
**1**	4.51	4.60	4.69	4.43	4.64	4.49	5.22	4.66
**2**	3.48	3.59	3.95	3.51	3.89	3.69	3.57	3.29
**3**	3.61	3.66	**4.06**	**3.70**	3.88	**3.82**	3.82	3.63
**4**	3.87	3.91	3.75	4.09	**3.92**	4.16	3.63	**3.64**
**5**	3.56	ND	3.53	3.58	3.59	ND	3.94	3.59
**6**	ND	3.74	3.95	3.69	3.98/3.85	3.97,3.85	ND	3.93

(b) ^{1}H assignments for Fuc residues of DF-*novo*-Hepta (in ppm)

	**VII** (present study)	**VII** (reference data)	**IX** (present study)	**IX** (reference data)
**1**	5.08	5.09	5.02	5.03
**2**	3.70	3.70	3.80	3.80
**3**	3.89	3.88	3.88,	3.88
**4**	3.78	3.78	3.79	3.79
**5**	4.81	4.81	4.86	4.87
**6**	1.16	1.15	1.16	1.18

Glycosylation positions assigned by HMBC and ROESY cross-peaks in bold; *indicates that assignments may be reversed.
Fuc positions in italic. Assignments for all Gal H5, C5 are tentative. ND: not determined.
References: Kogelberg et al: *Eur J Biochem*, 2004, 271, 1172-86; Gronberg et al. *Arch Biochem Biophys*, 1992, 296, 597-610

(a) HPLC system

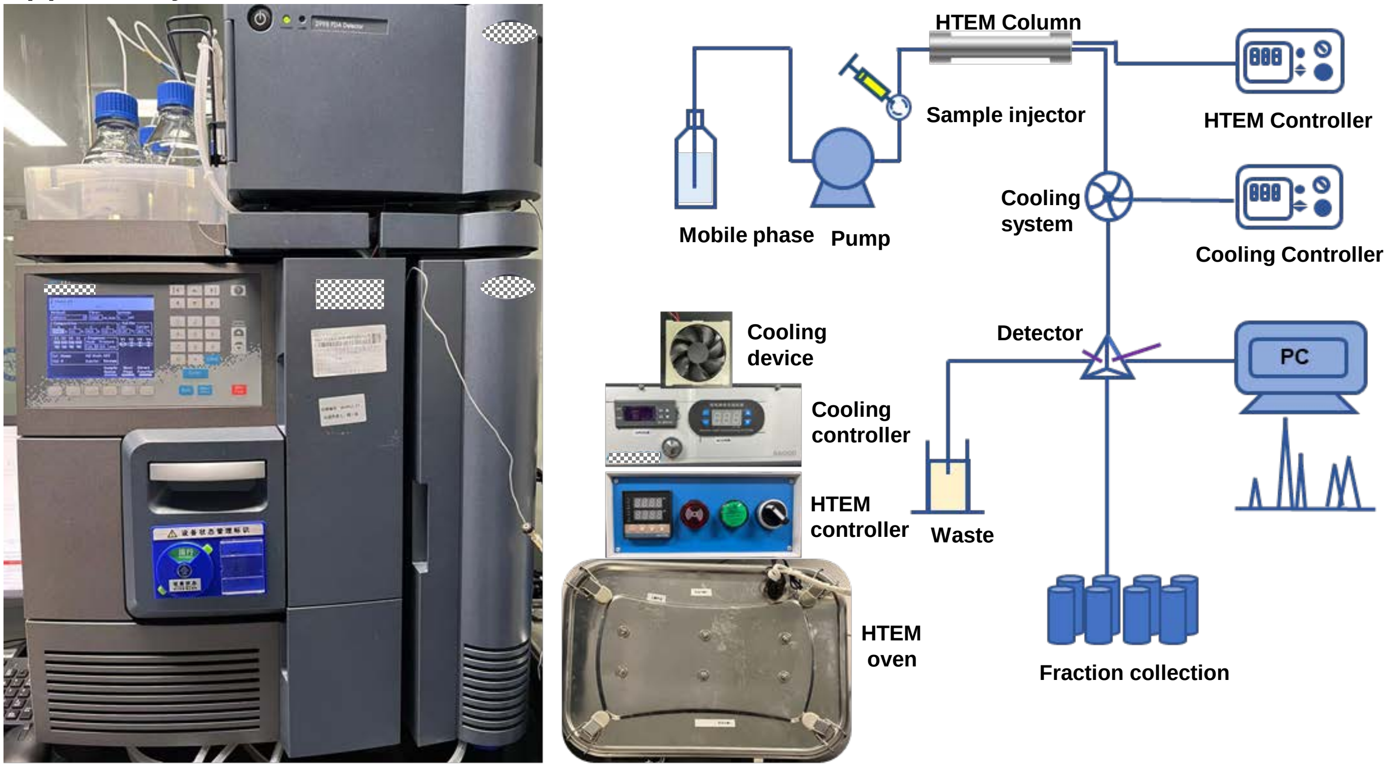

(b) High temperature oven

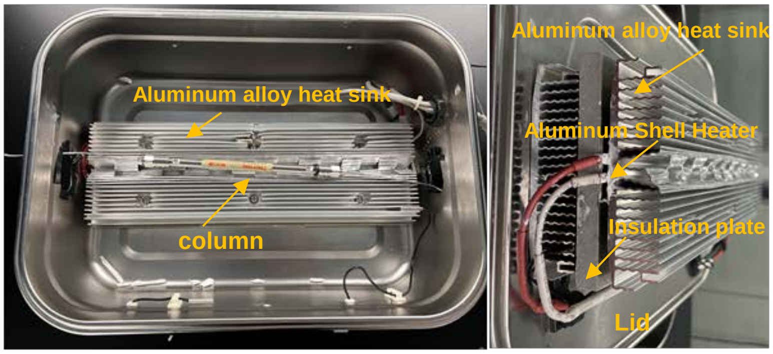

Inside view Side view

(c) Post-column cooling device

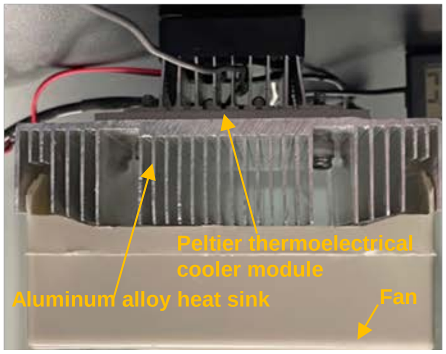

Top view

Figure S1. **Ultra-high temperature HPLC system.** (**a**) Modular components and layout of the ultra-high temperature HPLC system; (**b**) Inside and side view of the internal elements of the ultra-high temperature oven; (**c**) Post-column cooling device.

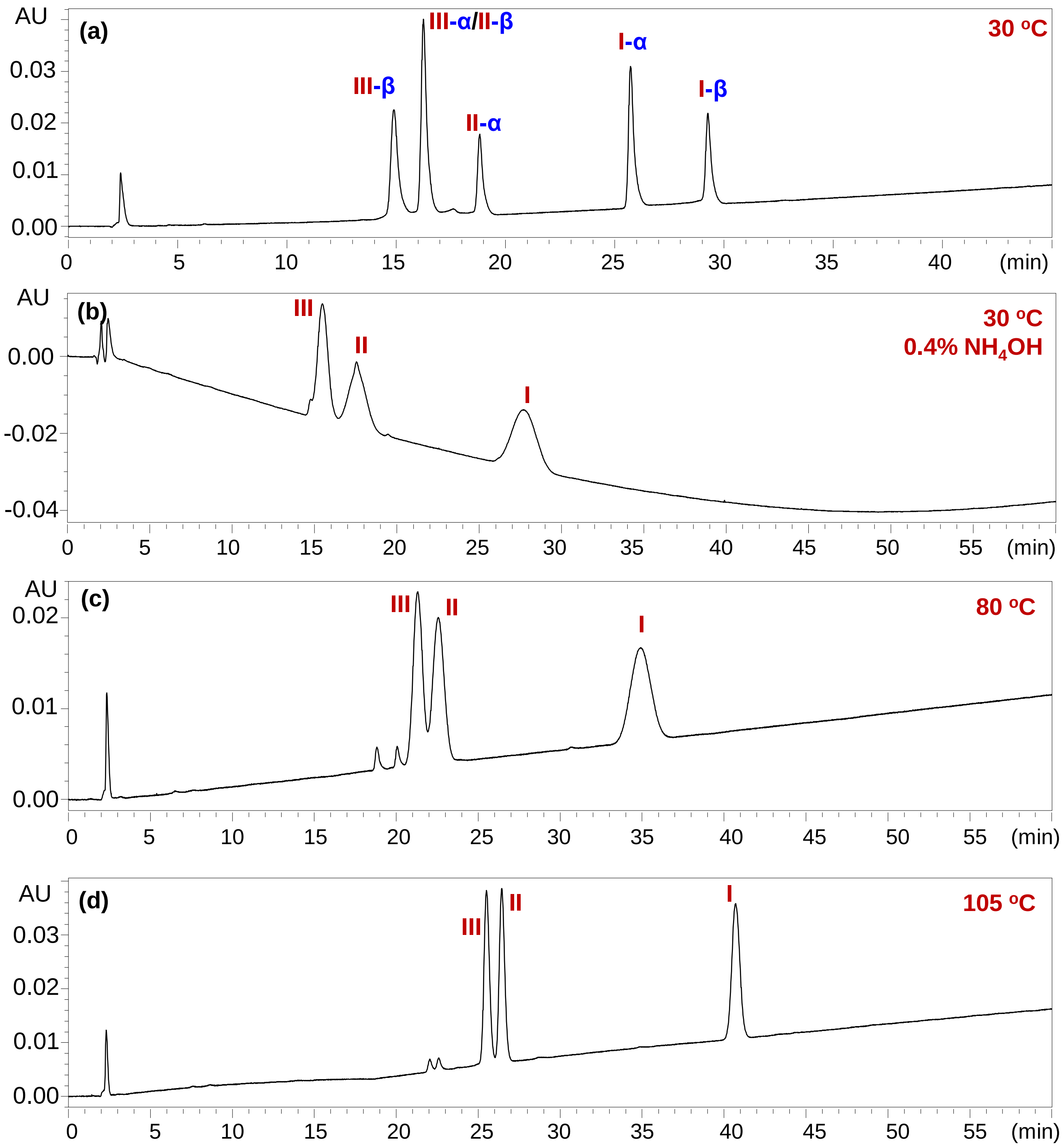

Figure S2. Optimization of PGC-HPLC conditions using a standard mixture of LNFP I, II and III. (**a**) Conventional temperature at 30 ℃ with ACN/H_2O gradient elution. (**b**) Conventional temperature at 30 ℃ with ACN/H_2O containing 0.4% NH_4OH gradient elution. (**c**) High temperature at 80 ℃ with ACN/H_2O gradient elution. (**d**) Ultra-high temperature at 105 ℃ with ACN/H_2O gradient elution. Note that AU is UV absorbance unit in HPLC analysis and 1 AU=1000 mA.

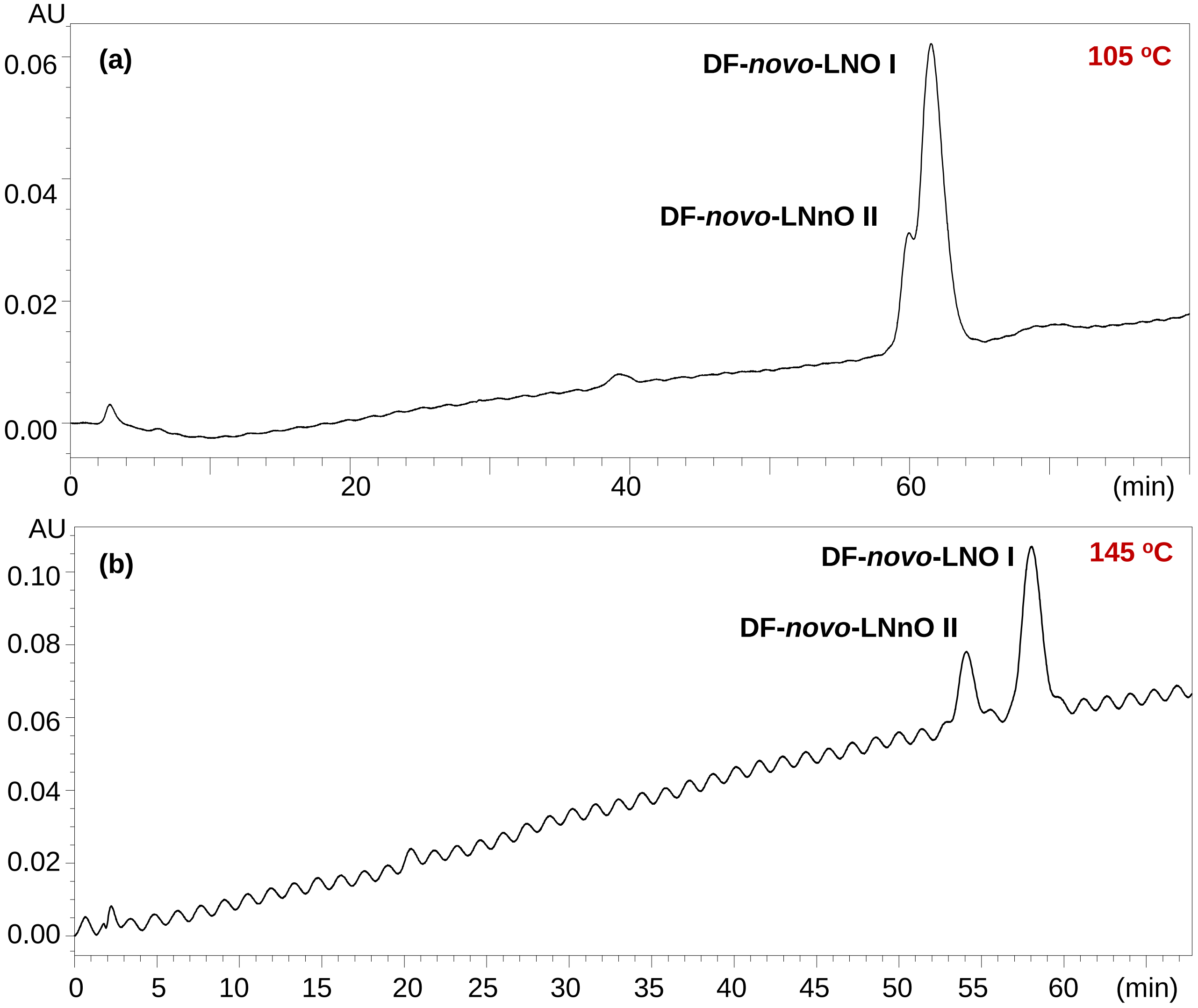

Figure S3. Optimization of temperature for PGC-HPLC separation of DF-*novo*-LNnO II and DF-*novo*-LNO I. (**a**) A high 105 ℃ with ACN /H_2O as the mobile phase was employed for PGC separation. (**b**) A ultra-high 145 ℃ with ACN /H_2O as the mobile phase was employed on PGC separation.

(a) ACN/H_2O containing 5 mM NH_4OAc

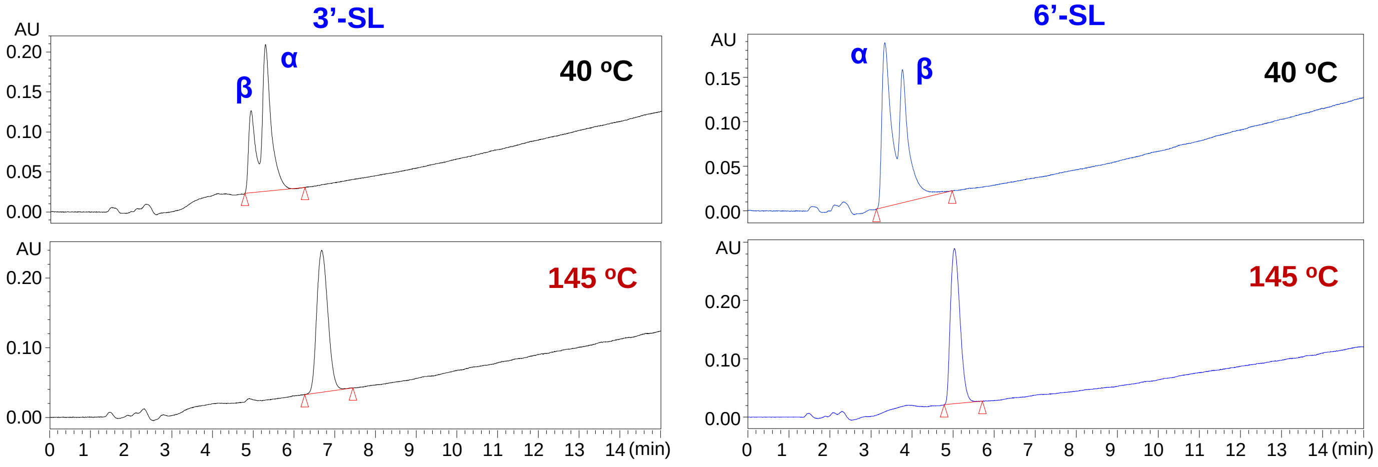

(b) ACN/H_2O containing 0.1% formic acid

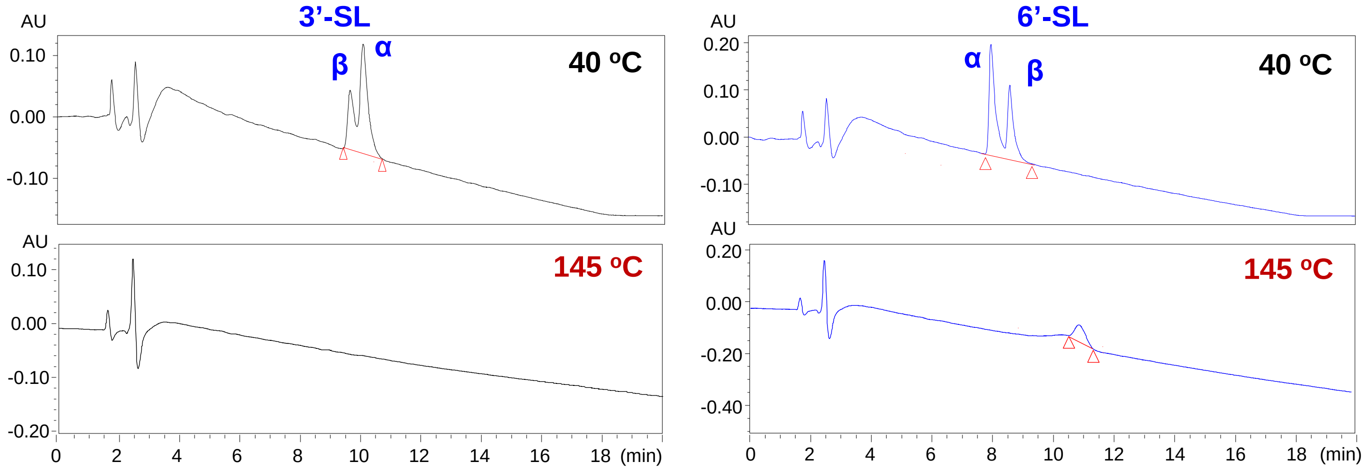

(c) Calculated stabilities of sialylated and neutral HMOs under ultra-high temperature conditions

	Solvent & Additive	HMO	Peak area (low T)	Peak area (high T)	Peak area ratio (high/low T)
**Sialyl**	ACN/H_2O NH_4OAc (5 mM)	3'-SL	4,000K (40 ºC)	3,765K (145 ºC)	0.94
		6'-SL	4,692K (40 ºC)	4,350K (145 ºC)	0.93
	ACN/H_2O Formic (0.1%)	3'-SL	4,832K (40 ºC)	0K (145 ºC)	0.00
		6'-SL	5,715K (40 ºC)	1,396K (145 ºC)	0.24
**Neutral**	ACN/H_2O	LNFP I	903K (30 ºC)	877K (110 ºC)	0.97

Figure S4. Stabilities of sialylated and neutral HMOs under different ultra-high temperature conditions using 3'-SL, 6'-SL and LNFP I as the test samples. In the solvent system ACN/H_2O, 5 mM NH_4OAc (**a**) or 0.1% formic acid (**b**) was included. Each experiment was repeated three times, and the averaged peak areas are listed (**c**).

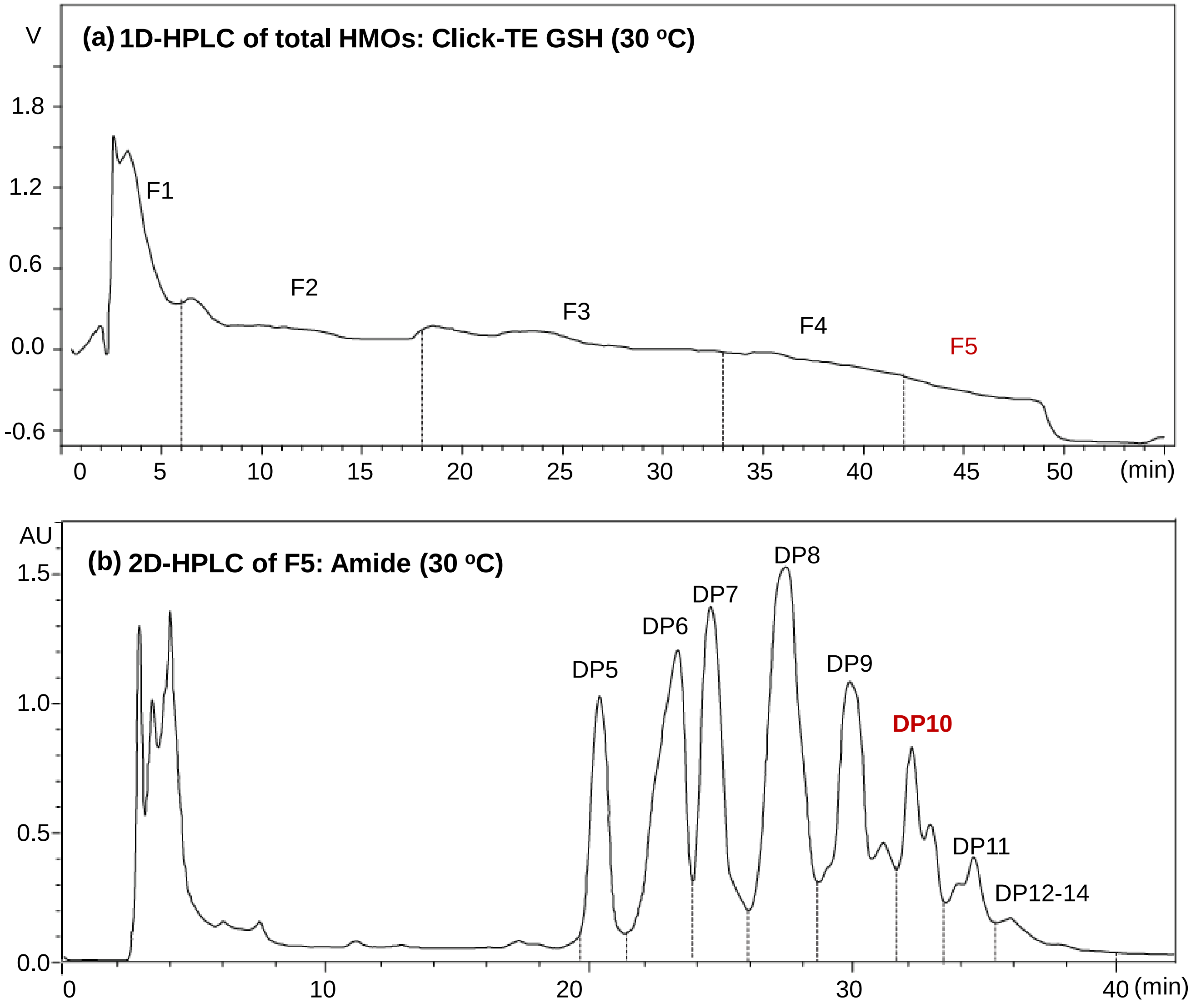

Figure S5. Group separation of HMOs based on two-dimensional hydrophilic chromatography. (**a**) Initial fractionation on a Click-TE GSH column. (**b**) Fraction F5 was further fractionated by HPLC on an amide column.

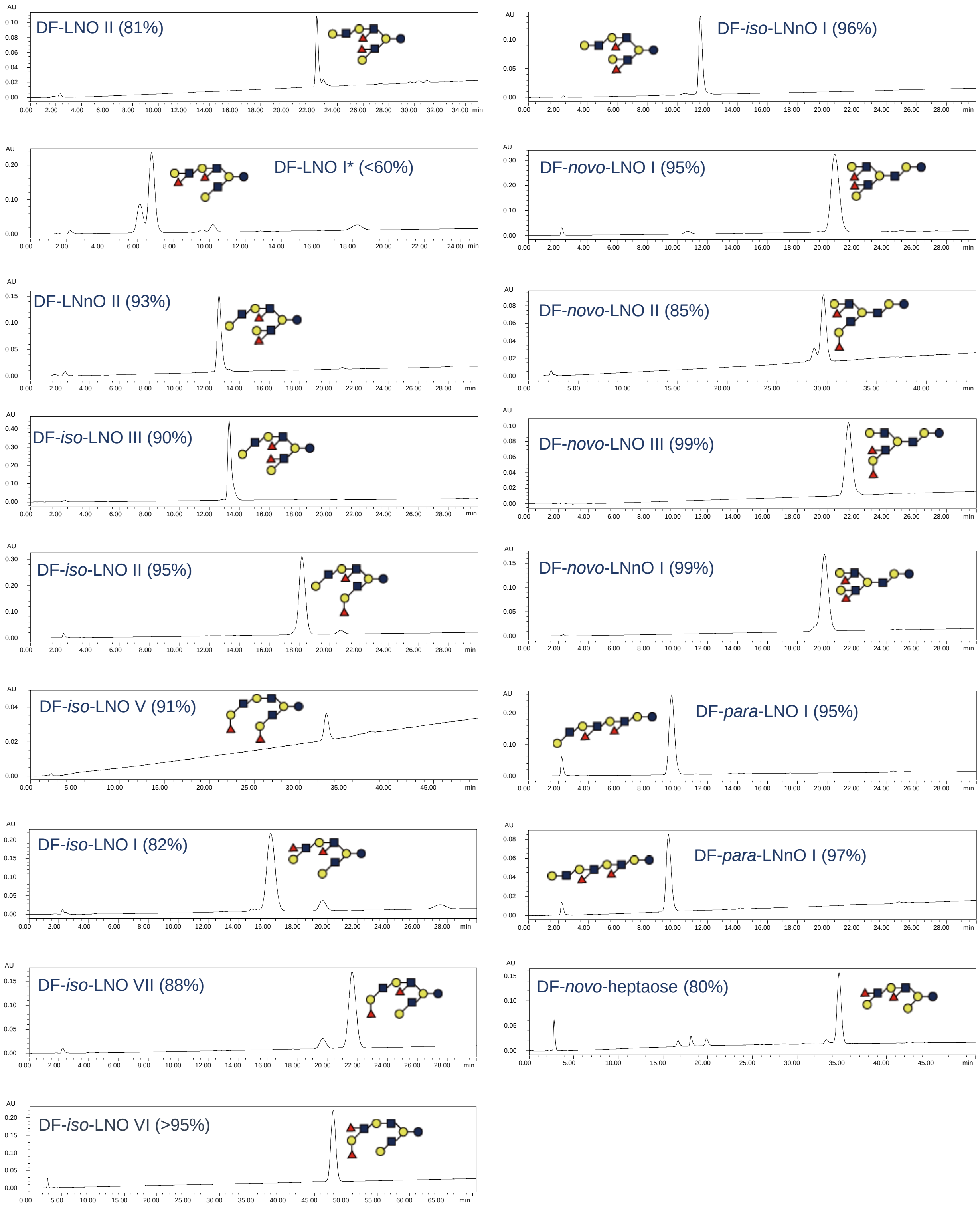

Figure S6. Purity analysis of 17 isolated HMOs by analytical scale PGC-HPLC .

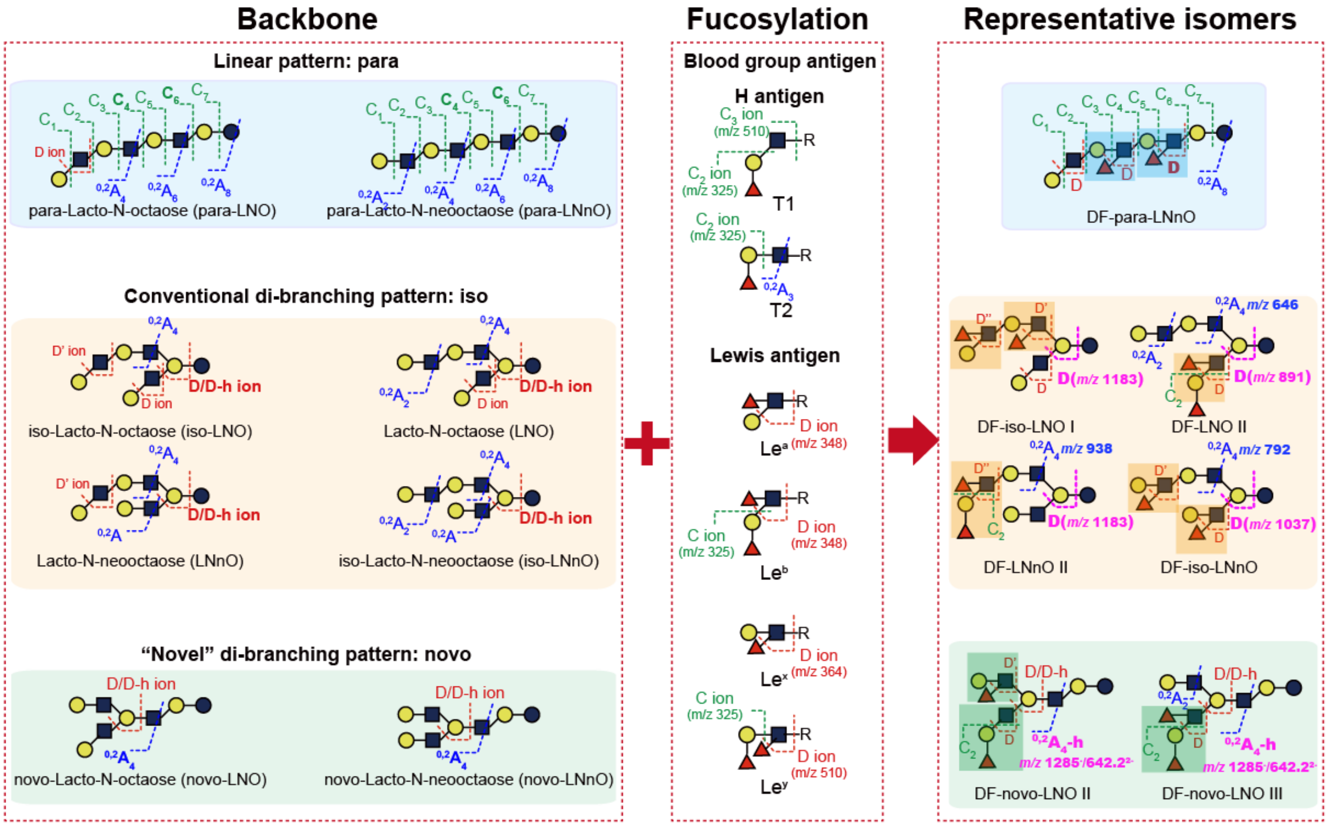

Figure S7. Workflow for ESI-MS^n analysis of difucosylated HMO isomers with octasaccharide backbones.

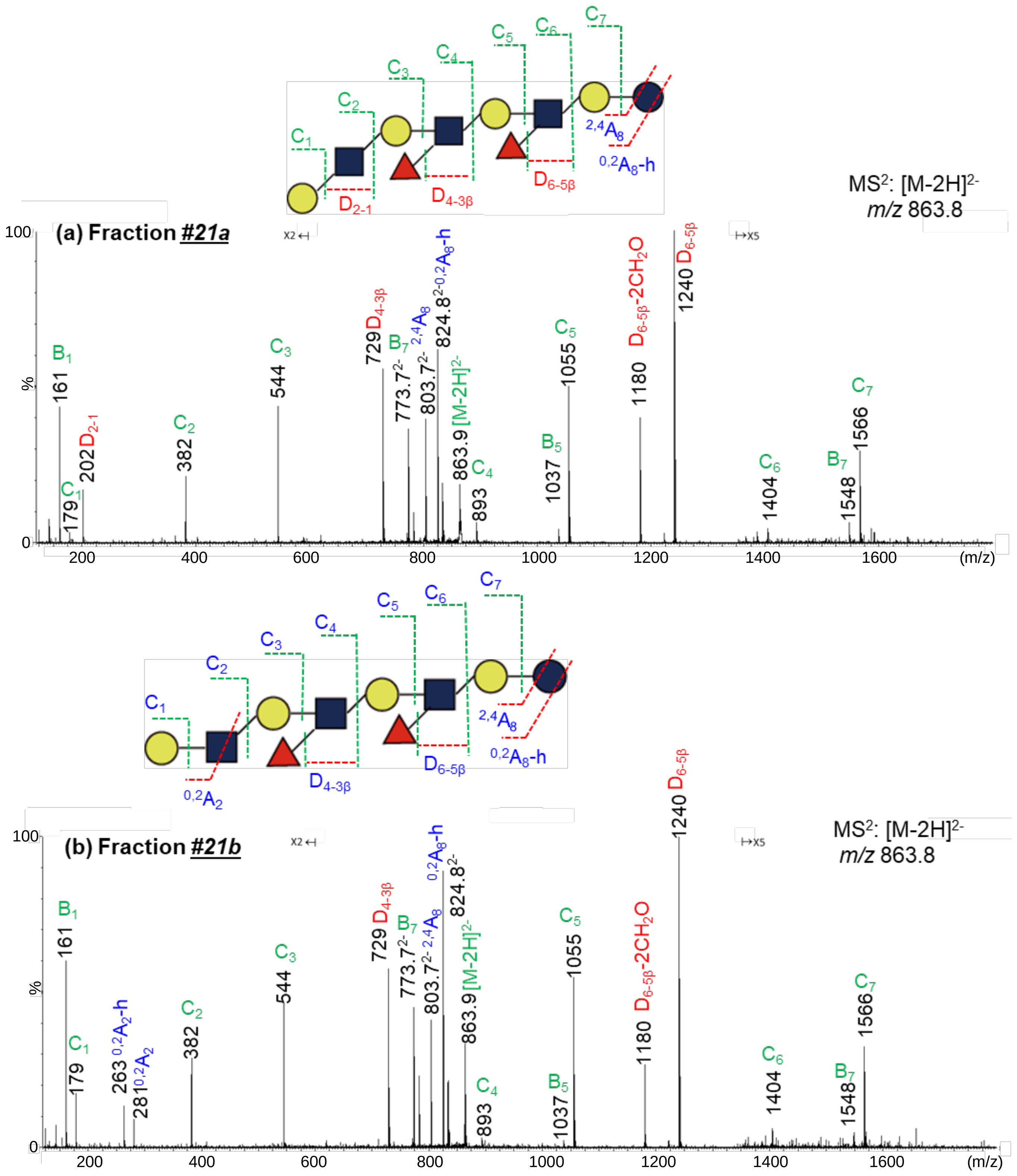

Figure S8. Negative-ion MS^2 of fraction #21a (DF-*para*-LNO I) and fraction #21b (DF-*para*-LNnO I). (**a**) MS^2 of #21a $[M-2H]^{2-}$ at *m/z* 863.8; (**b**) MS^2 of #21b $[M-2H]^{2-}$ at *m/z* 863.8.

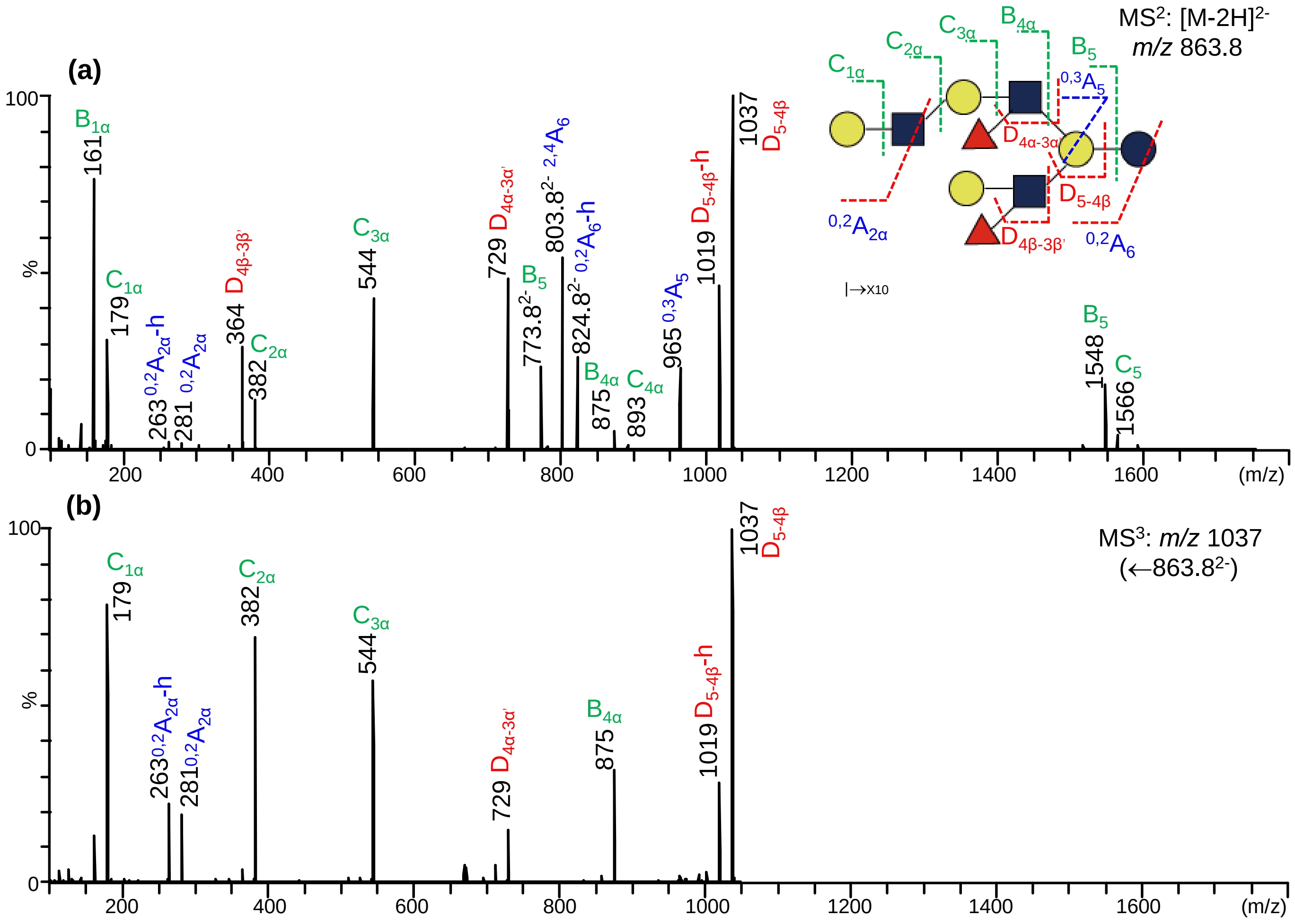

Figure S9. Negative-ion MS^n of fraction #24b (DF-*iso*-LNnO I). (**a**) MS^2 of $[M\text{-}2H]^{2-}$ at *m/z* 863.8 and (**b**) MS^3 of $D_{5\text{-}4\beta}$ at *m/z* 1037.

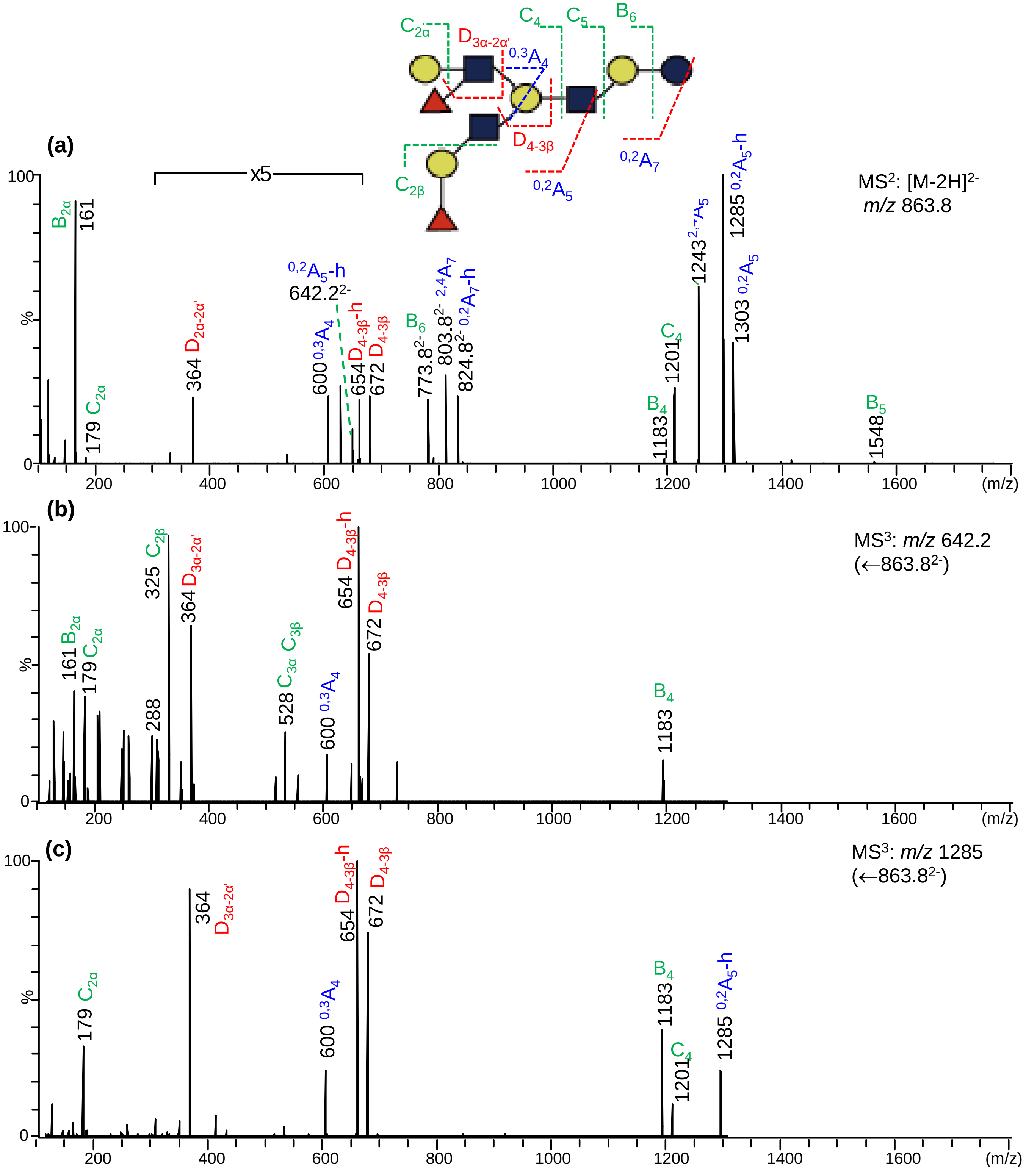

Figure S10. Negative-ion MS^n of fraction #52b (DF-*novo*-LNO II). (**a**) MS^2 of [M-2H]$^{2-}$ at *m/z* 863.8; (**b**) MS^3 of $^{0,2}A_4$-h [M-2H]$^{2-}$ at *m/z* 642.2; (**c**) MS^3 of $^{0,2}A_4$-h [M-H]$^-$ at *m/z* 1285.

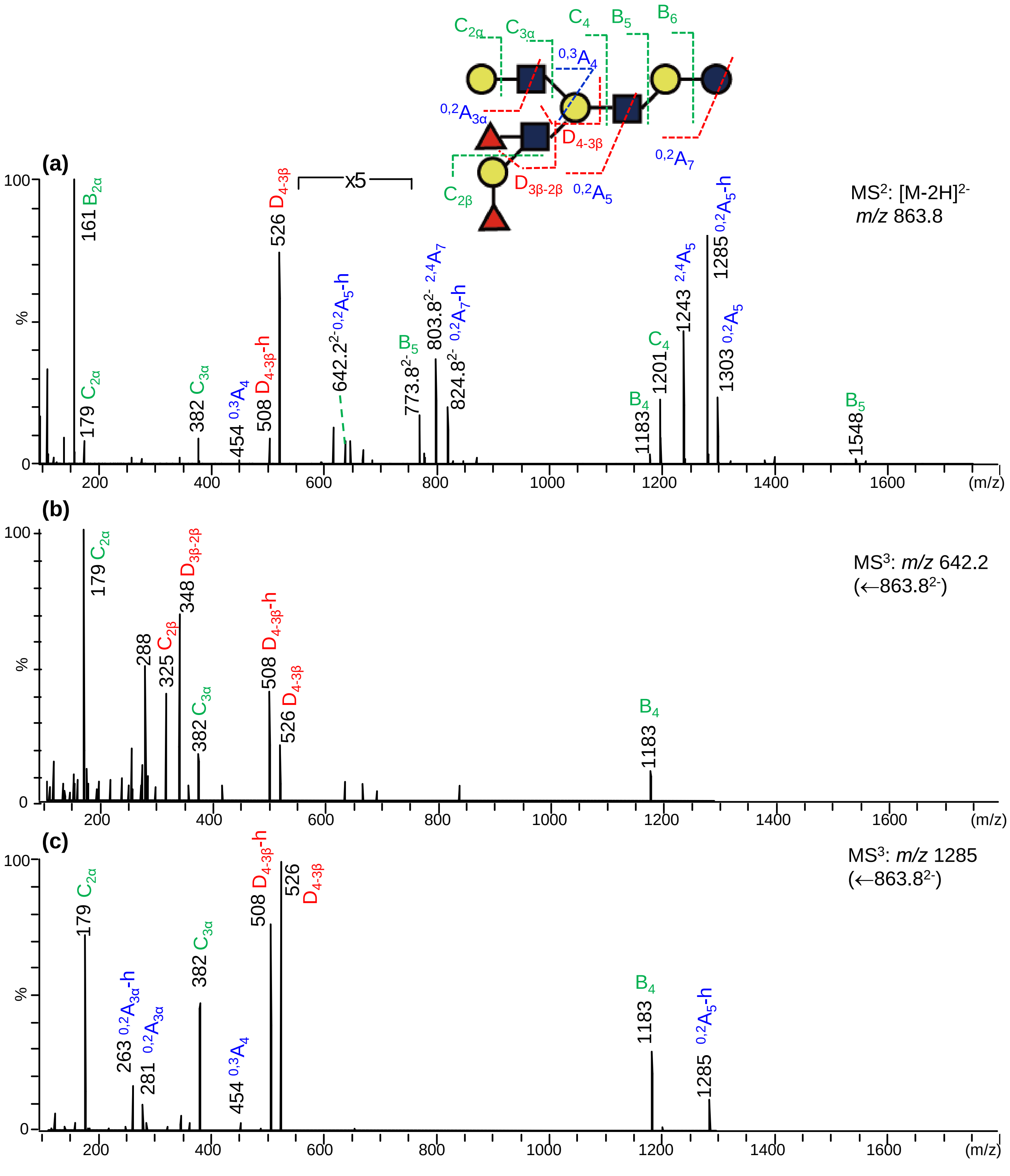

Figure S11. Negative-ion MS^n analysis of fraction #46d (DF-*novo*-LNO III). (**a**) MS^2 of [M-2H]$^{2-}$ at *m/z* 863.8; (**b**) MS^3 of $^{0,2}A_4$-h [M-2H]$^{2-}$ at *m/z* 642.2; (**c**) MS^3 of $^{0,2}A_4$-h [M-H]$^-$ at *m/z* 1285.

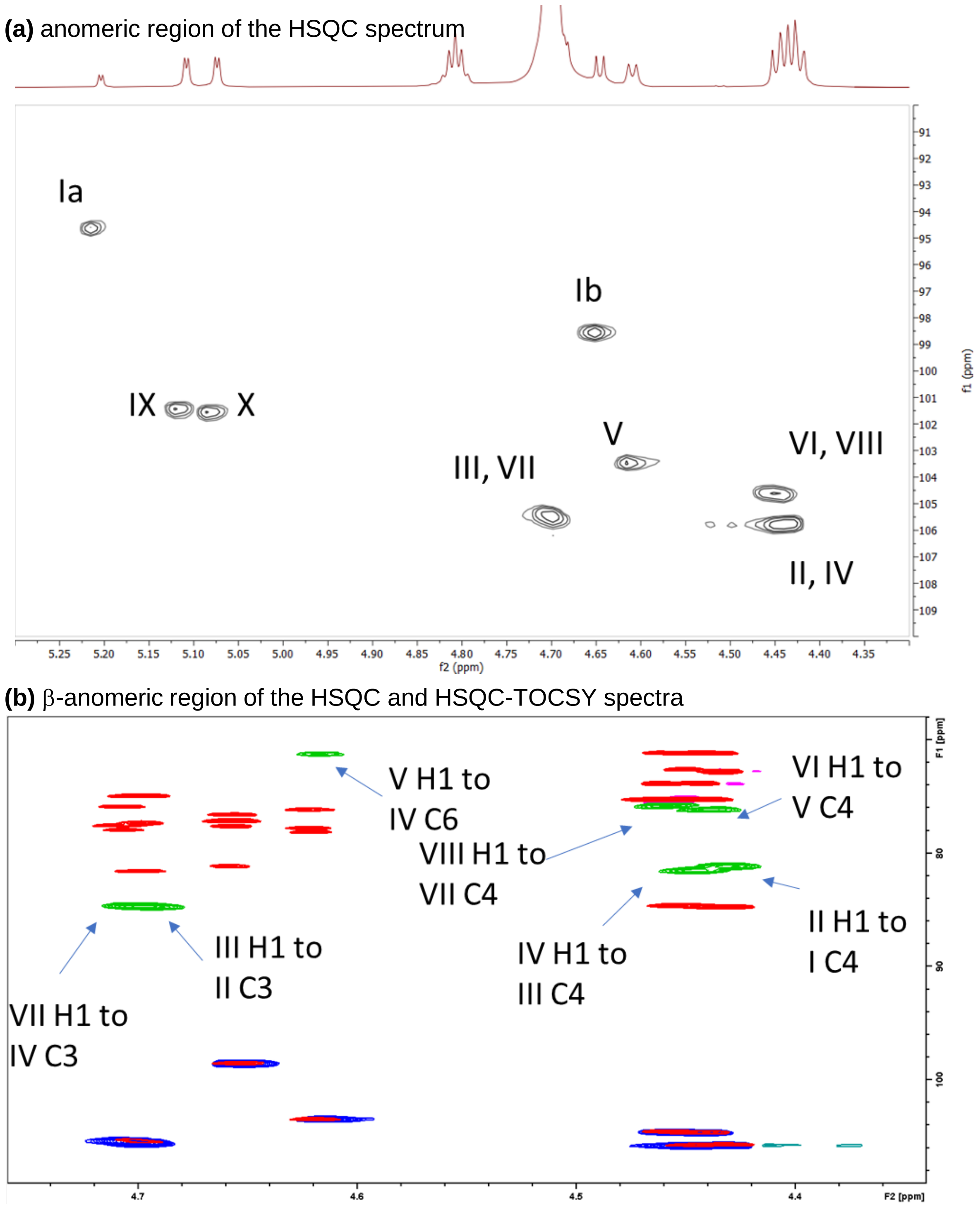

Figure S12. NMR spectra of #42b (DF-*novo*-LNnO I). (a) The anomeric region of the HSQC spectrum of DF-*novo*-LNnO I, showing cross-peaks characteristic of α-anomeric residues for Fuc residues IX and X, and the reducing end Glc α-anomer. The remaining β-anomeric cross peaks are assigned to Gal, GlcNAc and reducing end β-Glc residues. Some signals overlap, e.g. the anomeric cross-peaks from GlcNAc residues III and VII, and Gal residues VIII, VI, IV and II. **(b)** Expansion of the β-anomeric region of the HSQC (blue) and HSQC-TOCSY (red) spectra of DF-*novo*-LNnO I, overlaid with the HMBC spectrum (green). The HMBC peaks illustrated are inter-residue cross-peaks between H1 of one residue and the carbon immediately across the glycosidic linkage, defining both sequence and linkage positions.

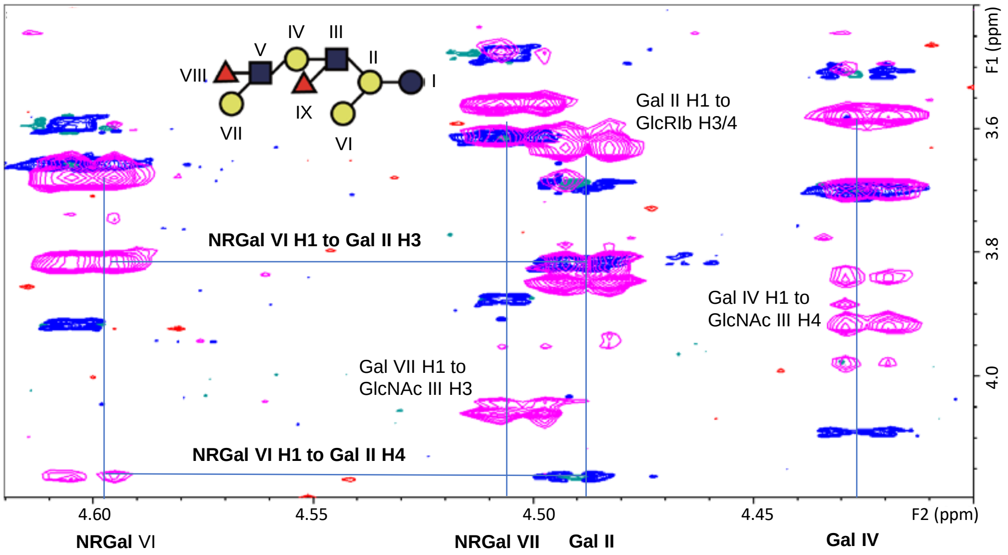

Figure S13. Overlaid TOCSY (blue) and ROESY (pink) NMR spectra of fraction #18a (DF-*novo*-Hepta). Expansion of the ^{1}H-^{1}H ROESY spectrum, showing inter-residue connectivity, particularly the two cross-peaks between Gal VI H1, and Gal II H3 and H4 (labelled in **bold**).

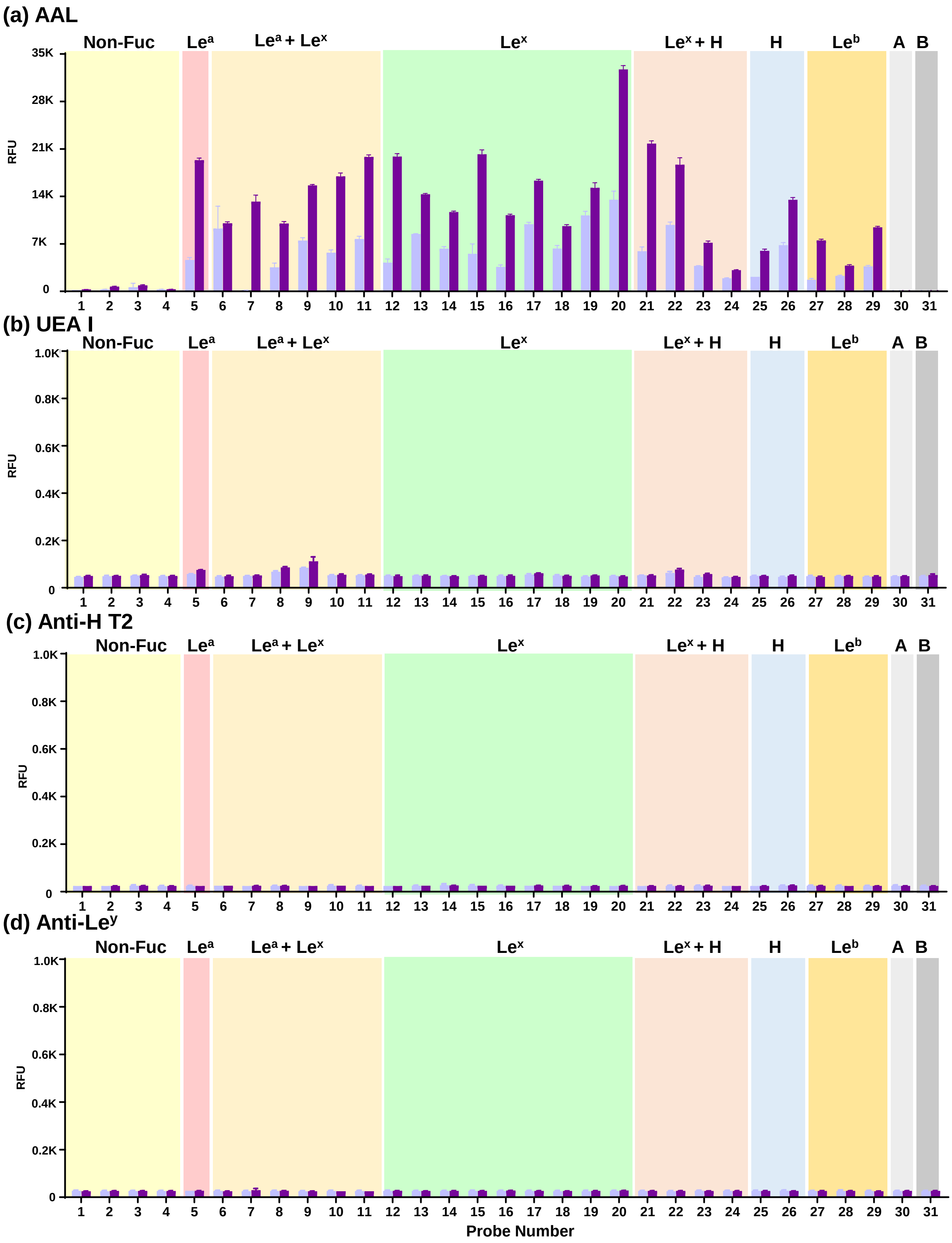

Figure S14 (a-d). Microarray binding of AAL (a), UEA I (b), anti- H type 2 (c), and anti-Le^{y} (d). Probes 1 to 4 of cream background: non-fucosylated HMOs; probe 5 of rose background: HMO with Le^{a}; probes 6 to 11 of pale yellow background: HMOs with both Le^{x} and Le^{a}; probes 12 to 20 with light green background: HMOs with Le^{x}; probes 21 to 24 of light orange background: HMOs with both Le^{x} and H; probes 25 and 26 of light blue background: HMOs with H; probes 27 to 29 of light gold background: HMOs with Le^{b}; probes 30 and 31 of light grey background: HMOs with blood group A and B epitope, respectively. The light and dark purple bars represent the probes with concentrations of 25 μM and 50 μM, respectively.

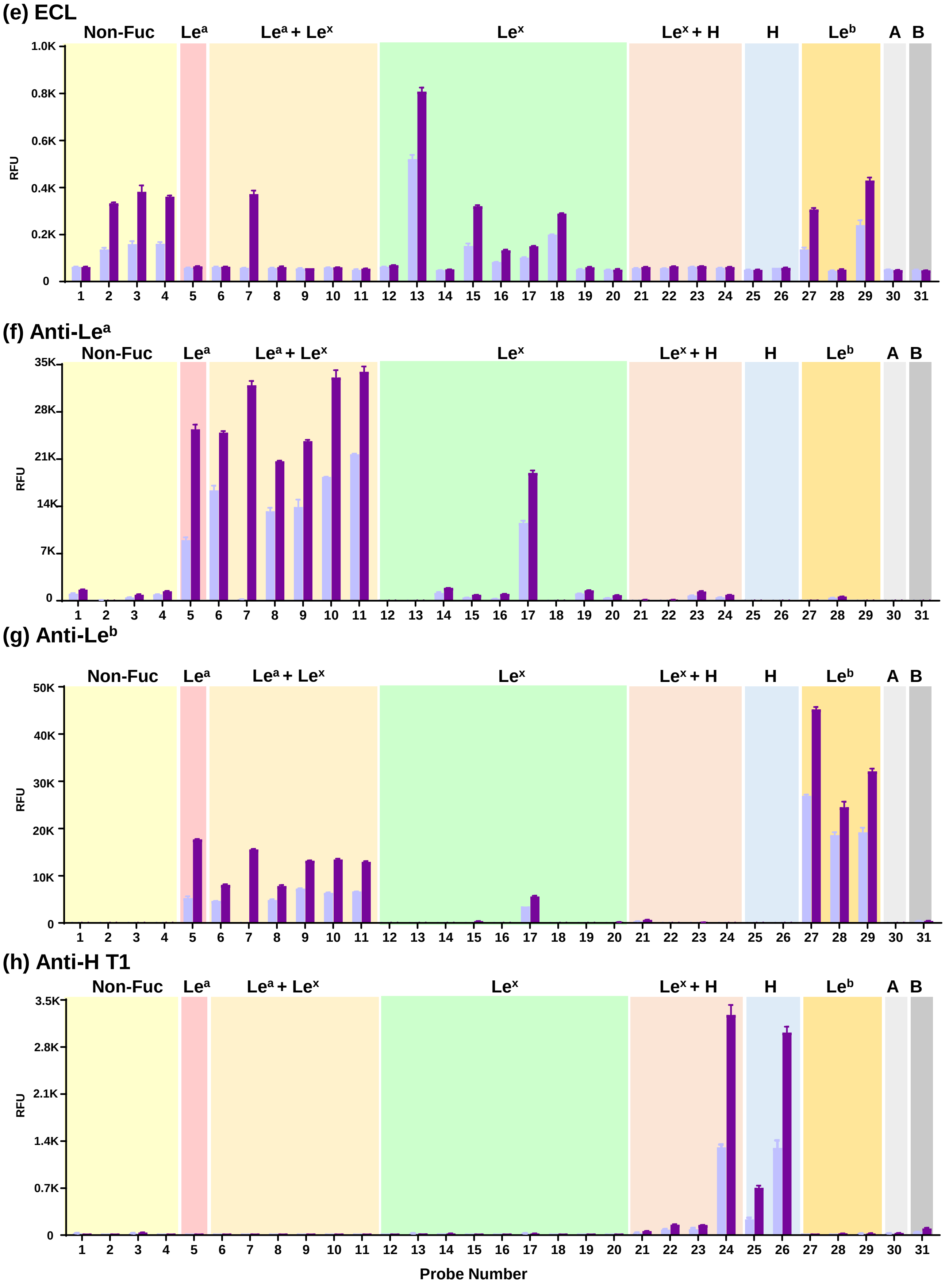

Figure S14 (e-h). Microarray binding of ECL (e), anti-Le^a (f), anti-Le^b (g), and anti-H type 1 (h).

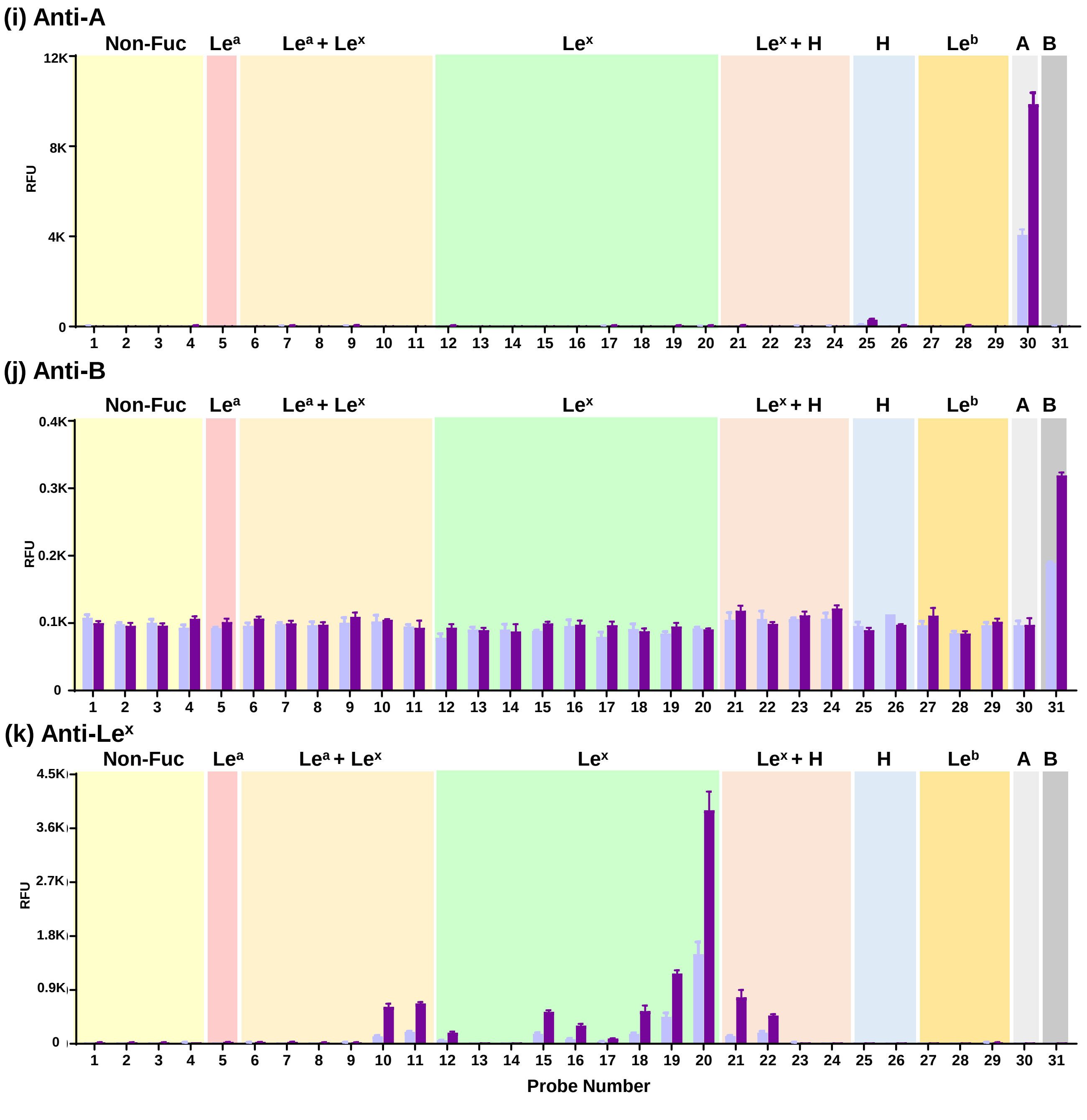

Figure S14 (i-k). Microarray binding of anti-blood group A (i), anti-blood group B (j) and anti-Le^{x} (k) .
